# Csep1^P^ protein from *Campylobacter concisus* induces a chemokine-dominant inflammatory state in macrophages and enhances proinflammatory response to gut bacteria

**DOI:** 10.1371/journal.ppat.1013951

**Published:** 2026-02-13

**Authors:** Christopher Yau Man Luk, Mohammad M. Rahman, Xiaotian Zhou, C. Mee Ling Munier, Fang Liu, Stephen M. Riordan, Anna Roujeinikova, Li Zhang

**Affiliations:** 1 School of Biotechnology and Biomolecular Sciences, University of New South Wales, Sydney, New South Wales, Australia; 2 Department of Microbiology, Monash University Monash Biomedicine Discovery Institute, Clayton, Victoria, Australia; 3 The Kirby Institute, University of New South Wales, Sydney, New South Wales, Australia; 4 Gastrointestinal and Liver Unit, Prince of Wales Hospital, Sydney, New South Wales, Australia; 5 Department of Biochemistry and Molecular Biology, Monash University, Clayton, Victoria, Australia; Centre National de la Recherche Scientifique, FRANCE

## Abstract

Translocation of *Campylobacter concisus* from the oral cavity to the intestinal tract is increasingly recognised as a contributor to inflammatory bowel disease (IBD). The *C. concisus* secreted protein Csep1 has emerged as a molecular marker of *C. concisus* strains associated with Crohn’s disease, a form of IBD. However, its structure and role in inflammation remain unknown. Here, we report the X-ray crystal structure of plasmid-encoded Csep1^P^ that reveals a unique α-helical fold with structural similarity to *Helicobacter pylori* cysteine-rich proteins HcpB and HcpC. Because HcpA, another Hcp family member, is known to affect monocyte differentiation, this structural similarity led us to hypothesise that Csep1^P^ may modulate monocyte differentiation and macrophage function. Transcriptomic analysis revealed that Csep1^P^ induced a chemokine-dominant inflammatory state in macrophages, M1-chem. Protein-level validation in both THP-1-derived and primary human macrophages confirmed this selective chemokine response. While Csep1^P^ alone did not upregulate proinflammatory cytokines, THP-1-derived macrophages pre-incubated with Csep1^P^ produced a higher level of proinflammatory cytokines in response to commensal *Escherichia coli*, which was validated on primary human macrophages. Furthermore, silencing the delta like canonical notch ligand 4 (*DLL4*) gene decreased the proinflammatory response of Csep1^P^-mediated macrophages to *E. coli*. Collectively, our data demonstrate that the structurally unique Csep1^P^ reprograms macrophage response, which provides a mechanistic link between *C. concisus* infection and Crohn’s disease pathogenesis, and identifies Csep1^P^ as a potential target for therapeutic intervention.

## Author summary

Crohn’s disease is a form of inflammatory bowel disease (IBD) that is a life-long chronic inflammation of the gastrointestinal tract. The causes of Crohn’s disease are complex and are not fully understood, but certain bacteria are thought to play a role. Our study focuses on *Campylobacter concisus*, a bacterium commonly found in the human oral cavity that can move to the gut and has been linked to Crohn’s disease. We investigated a *C. concisus* secreted protein called Csep1^P^, to better understand how it might affect the immune system. Using X-ray crystallography, we determined the unique 3D structure of this protein. We found that Csep1^P^ drives macrophages, a type of immune cell, into a state where they release specific signals that attract other immune cells. Although Csep1^P^ alone did not trigger inflammation, it “primed” macrophages to react much more strongly to commensal gut bacteria such as *Escherichia coli*. Our results suggest that Csep1^P^ may help explain how virulent *C. concisus* strains contribute to gut inflammation and highlight it as a potential target for new treatment for Crohn’s disease.

## Introduction

Crohn’s disease is a major form of inflammatory bowel disease (IBD), which encompasses a group of chronic inflammatory disorders of the gastrointestinal tract [1]. Crohn’s disease is most diagnosed in adolescents and young adults between 15 and 35 years, although it can occur at any age [1]. The aetiology of Crohn’s disease is multifactorial, involving a complex interplay between genetic predisposition, immune system dysregulation, environmental triggers and microbial factors [1]. Alterations in gut microbiota, characterised by reduced microbiota diversity, have been consistently observed in patients with Crohn’s disease as compared to healthy controls [2]. However, the specific bacterial genera or species reported to be decreased vary substantially across studies [2].

The hallmark of Crohn’s disease pathogenesis is chronic intestinal inflammation, driven by both innate and adaptive responses [1]. Among innate immune cells, macrophages play a central role in the initiation and perpetuation of inflammatory processes. Monocytes differentiate to macrophages in response to environmental stimuli, and naïve macrophages (M0) can polarise into proinflammatory M1 macrophages or anti-inflammatory M2 macrophages. Although the M1 and M2 framework represents two ends of macrophage activation, this binary classification is increasingly recognised as an oversimplification. Accumulating evidence indicates that macrophage activation exists along a spectrum of functional states shaped by distinct stimuli and tissue contexts [3]. In Crohn’s disease, particularly in the active stage of the disease, intestinal macrophages exhibit an inflammatory M1 type, which are thought to contribute to persistent tissue inflammation [4]. Recent single-cell transcriptomic profiling of colonic tissue from patients with Crohn’s disease has revealed substantial macrophage heterogeneity, with particularly big inter-patient differences in the myeloid compartment [5].

Current evidence supports the concept that Crohn’s disease arises from a dysregulated immune response to intestinal microbes including commensal bacteria [1]. Under physiological conditions, a balanced host-microbe relationship maintains intestinal homeostasis. In Crohn’s disease, this homeostasis is disrupted; however, the mechanisms underlying this breakdown remain incompletely understood.

*Campylobacter concisus* is a Gram-negative bacterium commonly found in the human oral cavity [6]. Translocation of *C. concisus* to various regions of the gastrointestinal tract has been associated with a range of inflammatory and pathological conditions [6]. For instance, detection of *C. concisus* in the oesophagus has been linked to Barrett’s oesophagus [7], while its presence in the stomach and intestines has been implicated in gastric inflammation [8,9], diarrhoeal disease [10], microscopic colitis [11], and IBD [12–16]. These observations have led to the suggestion that *C. concisus* may contribute to the development of a subset of IBD [17]. However, Nielsen *et al*. examined subsequent IBD development in patients with intestinal infection of *C. concisus* identified by bacterial culture, and did not observe an increased risk of IBD [18]. This discrepancy across studies suggests that the relationship between *C. concisus* and IBD may be context-dependent and that pathogenic potential may vary between strains, with only specific *C. concisus* strains contributing to IBD.

Liu *et al*. examined the bacterial genomes of *C. concisus* strains isolated from patients with IBD and healthy controls and identified the *csep1* gene and its association with active Crohn’s disease [19]. The *csep1* gene is located either on the *C. concisus* plasmid pICON (Csep1^P^) or on the chromosome (Csep1^C^) of a subgroup of *C. concisus* strains, and it encodes a secreted 24-kDa protein, Csep1 [19]. Notably, pICON has only been found in *C. concisus* strains isolated from patients with Crohn’s disease [19]. Despite this disease association, the Csep1 protein structure and its role in inflammation remain unknown.

In this study, we determined the protein structure of Csep1^P^ and investigated its impact on monocyte differentiation and macrophage activation. We found that Csep1^P^ adopts a novel protein structure and induced a previously unrecognised macrophage activation state, characterised by predominant chemokine production but not proinflammatory cytokines. Furthermore, Csep1^P^-primed macrophages exhibit an enhanced proinflammatory response to *Escherichia coli*. These findings provide insights into the mechanistic role of specific *C. concisus* molecules that may contribute to Crohn’s disease pathogenesis and identify Csep1^P^ as a potential target for therapeutic intervention.

## Results

### Csep1^P^ adopts a novel α-helical fold

To gain insights that could guide our investigation into the pathogenic mechanisms of Csep1^P^, we determined its structure by X-ray crystallography using the single-wavelength anomalous dispersion (SAD) technique at a resolution of 1.4 Å. Analysis of the crystal packing identified no stable quaternary structure, indicating that Csep1^P^ exists as a monomer in the crystal, consistent with its monomeric behaviour in solution [20].

**Fig 1:**
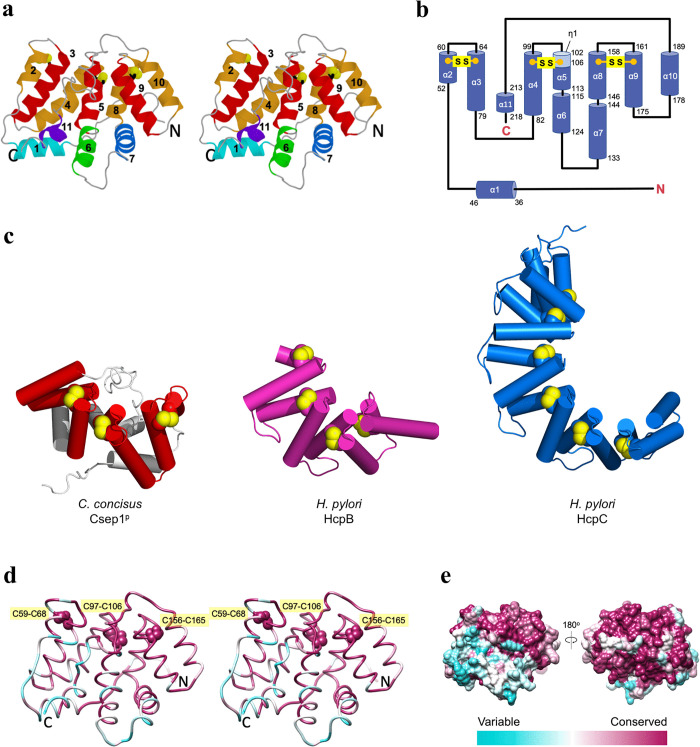
Structural analysis of *C. concisus* Csep1^P^. **(a)** Stereo representation of the Csep1^P^ structure. Helices that form the α-α-solenoid moiety are coloured in red and orange, while the extra-solenoidal helices are shown in green and blue hues. **(b)** Topology diagram of the secondary structure elements in Csep1^P^. The positions of the internal disulfide bonds stabilising the α-α-haipins (Cys-59/Cys-68, Cys-97/Cys-106 and Cys-156/Cys-165) are indicated. **(c)** Comparison of the crystal structure of *C. concisus* Csep1^P^ with *H. pylori* HcpB (PDB ID 1klx) and HcpC (PDB ID 1ouv). Note the similarity between the solenoid moiety of Csep1^P^ (coloured red) and the Sel1-like tetratricopeptide repeat (TPR) solenoid folds of HcpB and HcpC. Remarkably, the positions of the internal disulfide bonds stabilising the α-α-hairpins are highly conserved in these proteins, despite their very low overall sequence identity (see S1 Fig for structure superpositions). **(d)** Conserved residue clusters on the surface of the solenoid moiety of Csep1^P^. Stereoview of the protein backbone coloured according to the evolutionary conservation of each residue among 48 *C. concisus* strains. The colour gradient runs from cyan (not conserved) to magenta (fully conserved). Absolutely conserved internal disulfide bonds stabilising the α-α-hairpins are shown as spheres and labelled. **(e)** Molecular surface of *C. concisus* Csep1^P^, coloured according to the residue conservation.

Csep1^P^ folds into a single globular α-helical domain with a hemispherical shape measuring 54 × 57 × 45 Å (Fig 1a). The structure is composed of 11 α-helices, 7 of which (coloured red and orange in Fig 1a) are arranged in an α/α-solenoid configuration. The solenoid adopts a curved horseshoe-like structure, with each of the three α-α-hairpins stabilised by an internal disulfide bond (Cys-59/Cys-68, Cys-97/Cys-106 and Cys-156/ Cys-165; Fig 1a and 1b). The remaining four helices (coloured in green and blue hues in Fig 1a) form a small subdomain at one pole of the solenoid.

The comparison between the structure of *C. concisus* Csep1^P^ and all structures in the Protein Data Bank (PDB) using the DALI server [21] revealed that the fold of the solenoid moiety of the molecule is remotely similar to the Sel1-like tetratricopeptide repeat (TPR) fold [22,23] (S1 Table). However, the analysis of the amino acid sequence of Csep1^P^ using TPRpred (https://toolkit.tuebingen.mpg.de/tools/tprpred) identified no Sel1-like repeats in this protein [24]. Of particular interest are similarities to the *Helicobacter* cysteine-rich proteins HcpC [22] and HcpB [25]. Like in Csep1^P^, each of their α-α-hairpins is reinforced by an internal disulfide bridge. Although HcpC and the solenoid moiety of Csep1^P^ share only 17% amino acid sequence identity, they can be superimposed with a root mean square deviation (RMSD) of 3.1 Å for 99 aligned Cα atoms, indicating their significant overall similarity (S1 Fig). Figs 1c and S1 illustrate that not only HcpB, HcpC and the solenoid part of Csep1^P^ adopt a very similar fold, but the positions of the stabilising disulfide bonds are also highly conserved in these proteins. However, the similarities between Csep1^P^ and Hcps do not extend beyond the Csep1^P^ solenoid part – the extra-solenoidal helices α1, α6, α7, and α11 (shown in light grey) are unique to Csep1^P^.

### Conserved residues of Csep1^P^ map to its solenoid moiety

To analyse the Csep1^P^ residue conservation across different Csep1^P^ strains, we found 47 homologous amino acid sequences using PSI-BLAST search, aligned the sequences using COBALT [26] and used the alignment to generate a conservation profile with UCSF Chimera [27]. The backbone and the molecular surface representations of the structure of Csep1^P^ were then colour-coded according to the residue conservation scores (Fig 1d and 1e). This analysis showed that the cysteine residues that form disulfide bridges (Cys-59/Cys-68, Cys-97/Cys-106 and Cys-156/ Cys-165) stabilising the α-α-hairpins of the solenoid are absolutely conserved. It also clearly showed that the surface of the solenoid moiety of Csep1^P^ is highly conserved (Fig 1e), while most of the variable residues are mapped on the extra-solenoidal subdomain. The conservation of the disulfide-forming cysteine residues in the Csep1^P^ sequence, and in the structures of *C. concisus* Csep1^P^, *H. pylori* HcpB, and *H. pylori* HcpC (S1 Fig) suggests they are critical for the stability of the solenoid fold and control the angle between the adjacent helices of the α-α-hairpins. The TPR-like α/α-solenoid domains often mediate protein-protein interactions [28]. The conserved surface of the solenoid moiety of Csep1^P^ is likely important for its function and may represent the binding site(s) for (as yet unidentified) interacting proteins.

Partner proteins often bind to the concave surface of α/α-solenoids [28]. In the crystal structure of Csep1^P^, this solenoid surface is occupied by the protein’s C-terminus (S2 Fig). However, conservation analysis revealed a notable mismatch: highly variable C-terminus residues pack against highly conserved pockets on the solenoid surface. This pattern suggests that the C-terminus may shield a potential binding site in the crystal that could be exposed in solution.

### Gene expression analysis indicates that Csep1^P^ induced a chemokine-dominant macrophage activation

The protein structure analysis of Csep1^P^ revealed unexpected similarity to HcpB and HcpC. A previous study reported that HcpA, a member of the Hcp protein family, promoted THP-1 monocyte-to-macrophage differentiation [29]. We therefore assessed differentiation-associated transcriptional signatures and cell morphology following Csep1^P^ exposure, which did not support induction of a macrophage-like differentiation program under our experimental conditions, as indicated by the lack of expression in CD11b and CD68 markers, macrophage-like morphology, and enriched Gene Ontology (GO) terms (S3 Fig and S2 Table).

**Fig 2:**
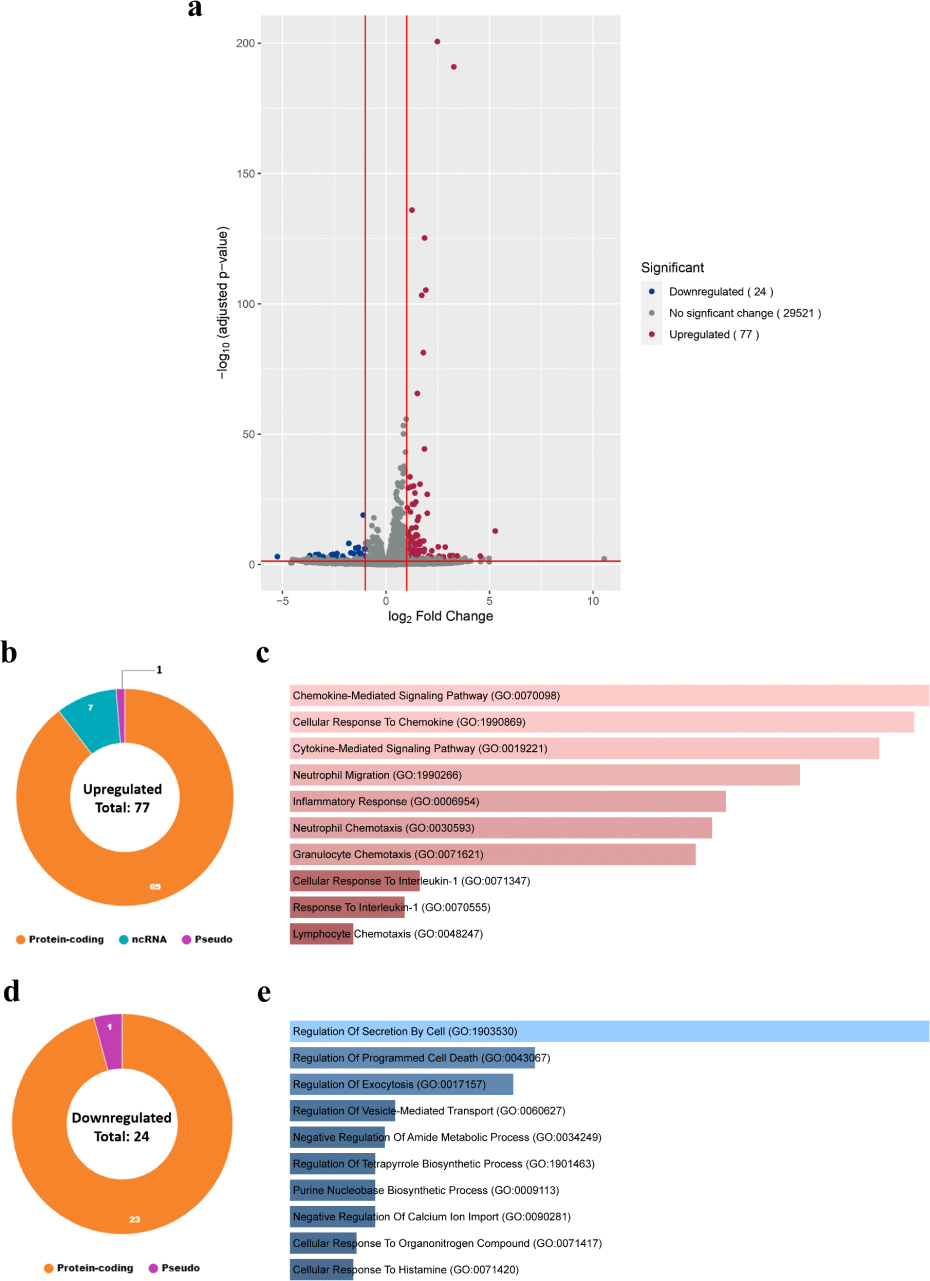
Transcriptomic analysis of global gene response to Csep1^P^ in THP-1-derived macrophages. THP-1-derived macrophages were incubated with Csep1^P^ for 24 hours and then subjected to RNA sequencing. **(a)** Volcano plot illustrating 29,622 transcripts identified in the RNA sequencing dataset, with 77 transcripts significantly upregulated (red) and 24 transcripts significantly downregulated (blue). Gene expression differences with adjusted *P*-value < 0.05 and log_2_ fold change ≤ -1 or ≥ 1 were considered statistically significant. The volcano plot was generated using the EnhancedVolcano package. **(b)** Of the 77 significantly upregulated genes, 69 were protein-coding, seven were non-coding RNAs, and one was a pseudogene. **(c)** Enriched Gene Ontology (GO) terms describing the biological processes associated with the 77 upregulated genes, sorted according to *P-*value. The top three enriched terms were “chemokine-mediated signalling pathway”, “cellular response to chemokine”, and “cytokine-mediated signalling pathway”. **(d)** Of the 24 significantly downregulated genes, 23 were protein-coding and one was a pseudogene. **(e)** Enriched GO terms of the 24 downregulated genes, sorted according to *P-*value. The top three enriched terms were “regulation of secretion by cell”, “regulation of programmed cell death”, and “regulation of exocytosis”.

In macrophages, Csep1^P^ induced an interesting activation pattern and showed an immunomodulatory effect. In THP-1-derived macrophages (M0 macrophages), Csep1^P^ significantly upregulated 77 transcripts, including 69 protein-coding genes, seven non-coding RNA and one pseudogene (Figs 2a, 2b, S4 and S5 and Table 1). The top GO term among upregulated genes was “chemokine-mediated signalling pathway” (Fig 2c). Csep1^P^ also significantly downregulated 24 transcripts, comprising 23 protein-coding genes and one pseudogene (Figs 2a, 2d, S4 and S5 and Table 1), with the top GO term for downregulated genes being “regulation of secretion by cell” (Fig 2e).

**Table 1:** Differentially expressed genes in THP-1-derived macrophages after incubation with Csep1^P^ protein from transcriptomic analysis.

Gene	Log2 FC	Padj	Gene ID	Type of Gene	Description
CCL4_2	5.278680	4.6E-11	6351	protein-coding	C-C motif chemokine ligand 4
PFDN6_4	4.555953	2.7E-02	10471	protein-coding	prefoldin subunit 6
CCL5_1	3.445406	2.4E-02	6352	protein-coding	C-C motif chemokine ligand 5
CXCL13	3.276492	1.7E-187	10563	protein-coding	C-X-C motif chemokine ligand 13
CCL4L1	3.240130	2.0E-02	388372	protein-coding	C-C motif chemokine ligand 4 like 1
LILRB2_3	3.089784	2.0E-02	10288	protein-coding	leukocyte immunoglobulin like receptor B2
PRTN3_1	2.864186	2.6E-05	5657	protein-coding	proteinase 3
KIFC1_2	2.781069	4.4E-02	3833	protein-coding	kinesin family member C1
LOC105377295	2.565088	2.9E-02	105377295	ncRNA	uncharacterised LOC105377295
COL8A1	2.525895	2.1E-05	1295	protein-coding	collagen type VIII alpha 1 chain
IGFBP3	2.482022	7.3E-197	3486	protein-coding	insulin like growth factor binding protein 3
C1QTNF1	2.215697	5.1E-04	114897	protein-coding	C1q and TNF related 1
PLEKHM1_2	2.132883	3.7E-02	9842	protein-coding	pleckstrin homology and RUN domain containing M1
RILP_1	2.055226	2.7E-02	83547	protein-coding	Rab interacting lysosomal protein
SHROOM3	1.997782	1.5E-17	57619	protein-coding	shroom family member 3
CCL19	1.996550	1.2E-24	6363	protein-coding	C-C motif chemokine ligand 19
TNFRSF9	1.924610	3.2E-102	3604	protein-coding	TNF receptor superfamily member 9
CXCL8	1.865548	4.1E-122	3576	protein-coding	C-X-C motif chemokine ligand 8
IL32	1.858675	1.2E-41	9235	protein-coding	interleukin 32
HLA-DQA1	1.850829	1.6E-04	3117	protein-coding	major histocompatibility complex, class II, DQ alpha 1
ANKRD22	1.824849	9.8E-04	118932	protein-coding	ankyrin repeat domain 22
TOP3A	1.820879	1.9E-07	7156	protein-coding	DNA topoisomerase III alpha
CXCL12	1.804907	2.3E-78	6387	protein-coding	C-X-C motif chemokine ligand 12
EBI3	1.729972	2.7E-100	10148	protein-coding	Epstein-Barr virus induced 3
MMP2-AS1	1.667893	4.2E-04	107984884	ncRNA	MMP2 antisense RNA 1
SIK1	1.655536	6.3E-04	150094	protein-coding	salt inducible kinase 1
TNFAIP6	1.647066	2.1E-28	7130	protein-coding	TNF alpha induced protein 6
CLDN1	1.631187	2.2E-07	9076	protein-coding	claudin 1
CCL8	1.629800	3.3E-06	6355	protein-coding	C-C motif chemokine ligand 8
CXCL1	1.575191	3.5E-16	2919	protein-coding	C-X-C motif chemokine ligand 1
MEOX2	1.556464	5.2E-03	4223	protein-coding	mesenchyme homeobox 2
IL18R1	1.546903	3.3E-04	8809	protein-coding	interleukin 18 receptor 1
CNTNAP1	1.523561	2.0E-09	8506	protein-coding	contactin associated protein 1
DLL4	1.519847	6.3E-15	54567	protein-coding	delta like canonical Notch ligand 4
FEZ1	1.517669	8.9E-63	9638	protein-coding	fasciculation and elongation protein zeta 1
FCAMR	1.497032	1.8E-03	83953	protein-coding	Fc alpha and mu receptor
C1QB	1.463266	1.1E-09	713	protein-coding	complement C1q B chain
GTF2H1	1.459655	2.7E-02	2965	protein-coding	general transcription factor IIH subunit 1
RPN2	1.448724	2.4E-12	6185	protein-coding	ribophorin II
TIFAB	1.444369	8.4E-22	497189	protein-coding	TIFA inhibitor
CCL20	1.444325	1.1E-06	6364	protein-coding	C-C motif chemokine ligand 20
CCL2	1.438738	1.6E-09	6347	protein-coding	C-C motif chemokine ligand 2
CXCL3	1.427649	4.2E-06	2921	protein-coding	C-X-C motif chemokine ligand 3
LOC105376714	1.415085	6.6E-04	105376714	protein-coding	SLC30A4 antisense RNA 1
IL1B	1.399793	3.9E-25	3553	protein-coding	interleukin 1 beta
LOC105375724	1.391491	3.9E-02	105375724	ncRNA	uncharacterised LOC105375724
CD80	1.391352	6.1E-09	941	protein-coding	CD80 molecule
ITGB8	1.391114	4.2E-06	3696	protein-coding	integrin subunit beta 8
CPAMD8	1.362663	6.1E-21	27151	protein-coding	C3 and PZP like alpha-2-macroglobulin domain containing 8
GLIS3	1.335333	9.8E-28	169792	protein-coding	GLIS family zinc finger 3
CACNG5	1.326814	3.2E-03	27091	protein-coding	calcium voltage-gated channel auxiliary subunit gamma 5
CLEC4E	1.322556	2.3E-04	26253	protein-coding	C-type lectin domain family 4 member E
PLEKHA1	1.321742	1.6E-02	59338	protein-coding	pleckstrin homology domain containing A1
PAK1IP1_1	1.290378	3.4E-02	55003	protein-coding	PAK1 interacting protein 1
ITGA1	1.280517	5.6E-21	3672	protein-coding	integrin subunit alpha 1
SOD2	1.256657	1.1E-132	6648	protein-coding	superoxide dismutase 2
MIR3142HG	1.253230	2.4E-07	107075116	ncRNA	MIR3142 host gene
MMP8	1.249504	4.5E-12	4317	protein-coding	matrix metallopeptidase 8
SERPINE2	1.203810	1.9E-27	5270	protein-coding	serpin family E member 2
SEMA7A_1	1.195217	6.7E-04	8482	protein-coding	semaphorin 7A (John Milton Hagen blood group)
PHF1_1	1.188522	2.0E-07	5252	protein-coding	PHD finger protein 1
G0S2	1.183534	4.8E-18	50486	protein-coding	G0/G1 switch 2
CXCL2	1.162926	6.1E-09	2920	protein-coding	C-X-C motif chemokine ligand 2
CRIM1	1.160303	3.9E-31	51232	protein-coding	cysteine rich transmembrane BMP regulator 1
MAPK10	1.121089	2.5E-03	5602	protein-coding	mitogen-activated protein kinase 10
POU2F2-AS1	1.120081	1.5E-05	100505622	ncRNA	long non-coding regulator of POU2F2
MAFF	1.089643	1.1E-10	23764	protein-coding	MAF bZIP transcription factor F
PTGER2	1.074938	3.1E-02	5732	protein-coding	prostaglandin E receptor 2
LOC124904743	1.071882	4.4E-27	124904743	ncRNA	uncharacterised LOC124904743
LOC107984475	1.069896	1.3E-05	107984475	ncRNA	uncharacterised LOC107984475
NCF1B	1.069448	9.1E-04	654816	pseudo	neutrophil cytosolic factor 1B (pseudogene)
IRAK2	1.063880	7.0E-10	3656	protein-coding	interleukin 1 receptor associated kinase 2
MMP1	1.044371	1.1E-04	4312	protein-coding	matrix metallopeptidase 1
GPR68	1.040779	3.4E-02	8111	protein-coding	G protein-coupled receptor 68
BIRC3	1.033185	1.3E-19	330	protein-coding	baculoviral IAP repeat containing 3
IDO1	1.022883	4.1E-02	3620	protein-coding	indoleamine 2,3-dioxygenase 1
SEMA3A	1.011619	2.2E-06	10371	protein-coding	semaphorin 3A
GPR89B	-1.025993	1.1E-04	51463	protein-coding	G protein-coupled receptor 89B
SEPTIN4	-1.090768	2.5E-02	5414	protein-coding	septin 4
GABRB2	-1.102198	6.5E-17	2561	protein-coding	gamma-aminobutyric acid type A receptor subunit beta2
ARC	-1.136733	4.3E-02	23237	protein-coding	activity regulated cytoskeleton associated protein
RETSAT_1	-1.207608	3.8E-02	54884	protein-coding	retinol saturase
SNCAIP	-1.211555	3.6E-03	9627	protein-coding	synuclein alpha interacting protein
IGLON5	-1.244578	4.7E-03	402665	protein-coding	IgLON family member 5
SHMT1	-1.304212	1.4E-03	6470	protein-coding	serine hydroxymethyltransferase 1
TBL3_1	-1.326445	3.4E-05	10607	protein-coding	transducin beta like 3
ROBO4	-1.459278	6.1E-05	54538	protein-coding	roundabout guidance receptor 4
LOC102724594	-1.563153	4.4E-03	102724594	protein-coding	U2 small nuclear RNA auxiliary factor 1 like 5
CIDEB	-1.699159	2.4E-03	27141	protein-coding	cell death inducing DFFA like effector b
XAGE1B	-1.794569	1.3E-06	653067	protein-coding	X antigen family member 1B
LOC124902436	-2.082312	4.2E-02	124902436	protein-coding	talanin
WHAMMP1_2	-2.085552	2.9E-02	100288615	pseudo	WHAMM pseudogene 1
RASA3_1	-2.365075	4.2E-03	22821	protein-coding	RAS p21 protein activator 3
HMOX2_1	-2.429701	4.7E-02	3163	protein-coding	heme oxygenase 2
SPATC1L_1	-2.555276	7.2E-03	84221	protein-coding	spermatogenesis and centriole associated 1 like
MCCC2_2	-2.637574	1.1E-02	64087	protein-coding	methylcrotonyl-CoA carboxylase subunit 2
TMEM14C_1	-3.041863	4.0E-02	51522	protein-coding	transmembrane protein 14C
APEX1_1	-3.260226	7.9E-03	328	protein-coding	apurinic/apyrimidinic endodeoxyribonuclease 1
GOLGA8J_2	-3.401399	1.0E-02	653073	protein-coding	golgin A8 family member J
TRIM27_2	-3.683670	1.9E-02	5987	protein-coding	tripartite motif containing 27
SCARA5	-5.244875	4.0E-02	286133	protein-coding	scavenger receptor class A member 5

THP-1-derived macrophages were incubated with Csep1^P^ (10 µg/ml) for 24 hours before RNA extraction and RNA sequencing.

Transcripts with adjusted *P*-value < 0.05 and log_2_ fold change ≤ -1 or ≥ 1 were considered differentially expressed genes.

**Fig 3:**
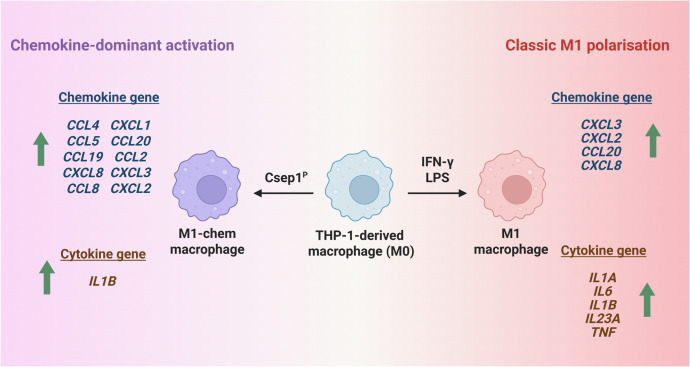
Comparison of upregulated M1 macrophage-associated chemokine and cytokine genes induced by Csep1^P^ and LPS. Csep1^P^ induced THP-1-derived macrophages (M0) into a chemokine-dominant activation state, designated here as M1-chem. M1-chem macrophages were characterised by the upregulation of a broad range of chemokine genes, including those typically induced in classical M1 macrophages stimulated with LPS plus IFN-γ, but lacked many hallmark proinflammatory M1 cytokines. Two sets of RNA-seq data from THP-1-derived macrophages were used for analysis: the Csep1^P^-induced gene expression data were generated in this study, while the LPS plus IFN-γ-induced gene expression data were obtained from public database [30]. Only genes significantly upregulated (adjusted *P*-value < 0.05, log2 fold change ≥ 1) were included for comparison. Created in BioRender. Luk, C. (2026) (https://BioRender.com/byr6efn) is licensed under CC BY 4.0.

Among inflammatory mediator genes, Csep1^P^ primarily induced chemokine gene expression rather than a broad classical proinflammatory cytokine profile. Specifically, Csep1^P^ upregulated ten chemokine genes and only one proinflammatory cytokine gene, *IL1B* (Fig 3 and Table 2). This gene expression profile differed markedly from the canonical M1 proinflammatory macrophage activation state induced by lipopolysaccharide (LPS) and interferon-gamma (IFN-γ), which is characterised by significant upregulation of proinflammatory cytokine genes *IL1A*, *IL1B*, *IL6*, *IL23A*, and *TNF* (Fig 3 and Table 2) [30]. We designated the Csep1^P^-induced macrophage state as M1-chem, where ‘chem’ denotes the predominant upregulation of chemokines.

**Table 2:** Upregulated chemokine and cytokine genes in THP-1-derived macrophages induced by Csep1^P^ compared to LPS + IFN-γ.

Csep1^P^			LPS + IFN-γ		
Chemokines	Log_2_ FC	Padj	Chemokines	Log_2_ FC	Padj
CCL4	5.278680	5E-11	CXCL3	9.239138	2E-09
CCL5	3.445406	2E-02	CCL1	6.685374	1E-07
CXCL13	3.276492	2E-187	CXCL2	5.461780	5E-03
CCL4L1	3.240130	2E-02	CCL20	3.474956	1E-65
CCL19	1.996550	1E-24	CCL3	3.015943	2E-09
CXCL8	1.865548	4E-122	CXCL8	2.536549	8E-08
CXCL12	1.804907	2E-78	CCL3L1	1.759345	1E-02
CCL8	1.629800	3E-06			
CXCL1	1.575191	4E-16			
CCL20	1.444325	1E-06			
CCL2	1.438738	2E-09			
CXCL3	1.427649	4E-06			
CXCL2	1.162926	6E-09			
Cytokines	Log_2_ FC	Padj	Cytokines	Log_2_ FC	Padj
IL32	1.858675	1E-41	IL1A	9.376056	3E-09
IL1B	1.399793	4E-25	IL6	8.227734	3E-06
			IL1B	4.598253	4E-35
			IL23A	4.016216	1E-19
			LIF	3.300077	9E-11
			TNF	2.322512	9E-13
			TNFSF15	2.095261	6E-05
			TNFSF10	1.567895	2E-02
			IL24	1.301356	1E-02

Genes highlighted in grey represents M1-associated genes.

Only genes significantly upregulated (adjusted *P*-value < 0.05, log2 fold change ≥ 1) were included.

Genes for Csep1^P^ column were extracted from this study (see Table 1 for full list).

Genes for LPS + IFN-γ column were extracted from a study conducted by Panahipour, L. *et al*. [30].

### Validation of chemokine and proinflammatory cytokine protein responses support a chemokine-dominant macrophage activation state induced by Csep1^P^

**Fig 4:**
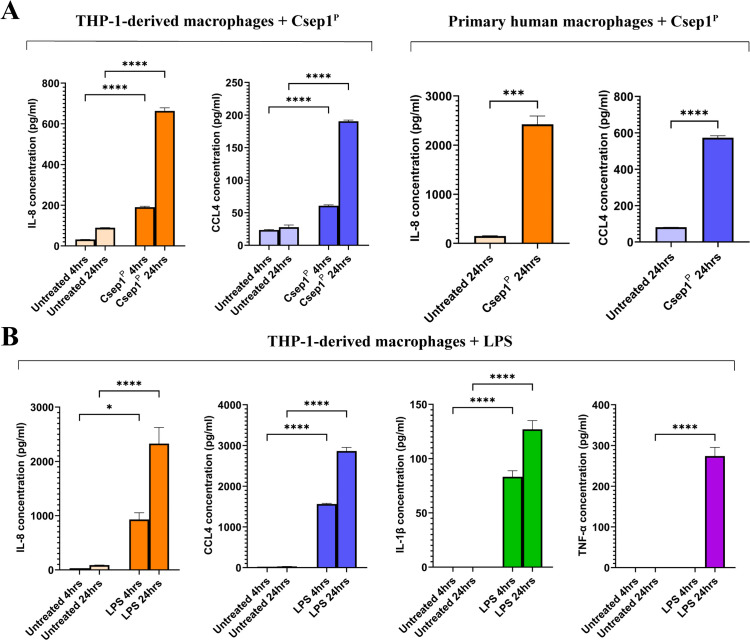
Csep1^P^ and LPS induced chemokines and proinflammatory cytokines in macrophages. The levels of chemokines IL-8 and CCL4, and proinflammatory cytokines IL-1β and TNF-α in the supernatants of THP-1-derived macrophages stimulated with PMA for 2 days and primary human macrophages following incubation with Csep1^P^ (10 μg/ml) or LPS (100 ng/ml) were measured using ELISA. **(a)** In both THP-1-derived macrophages and human primary macrophages, Csep1^P^ significantly increased the production of IL-8 and CCL4, while IL-1β and TNF-α were below the limit of detection. **(b)** LPS significantly increased the production of all four cytokines in THP-1-derived macrophages. Statistical analysis was performed using one-way analysis of variance (ANOVA) with Tukey’s post hoc test or two-tailed unpaired t-test. Bars represent the mean of triplicate experiments ± SEM. *** *P* < 0.001, **** *P* < 0.0001.

To validate the RNA-seq findings, we quantified representative chemokines (IL-8/CXCL8 and CCL4) and proinflammatory cytokines (IL-1β and TNF-α) by ELISA in THP-1-derived macrophages and primary human macrophages treated with Csep1^P^ or LPS. Consistent with the transcriptomic data, Csep1^P^ induced a robust increase in chemokine production, while proinflammatory cytokines were not detected (Fig 4).

In THP-1-derived macrophages, Csep1^P^ induced a significant increase in IL-8 secretion at both 4 hours (190.4 ± 3.5 pg/ml versus 31.7 ± 0.2 pg/ml in controls; *P* < 0.0001) and 24 hours (663.6 ± 12.4 pg/ml versus 89.2 ± 0.8 pg/ml; *P* < 0.0001; Fig 4a). CCL4 production also showed a significant increase at both time points: 60.8 ± 1.0 pg/ml (versus 23.6 ± 0.6 pg/ml) after 4 hours and 190.5 ± 1.5 pg/ml (versus 27.8 ± 2.7 pg/ml) after 24 hours (*P* < 0.0001 for both; Fig 4a). Both IL-1β and TNF-α were below the limit of detection.

In primary human macrophages differentiated from peripheral blood mononuclear cells (PBMCs; S6 Fig), Csep1^P^ treatment for 24 hours resulted in a significant upregulation of IL-8 (2,423 ± 168.7 pg/ml compared to 149.1 ± 7.5 pg/ml in controls; *P* < 0.001; Fig 4a). Similarly, CCL4 production was significantly higher (573.1 ± 10.9 pg/ml compared to 80.8 ± 0.4 pg/ml in controls; *P* < 0.0001; Fig 4a). Both IL-1β and TNF-α were below the limit of detection.

LPS significantly increased the production of both chemokines IL-8 and CCL4, and proinflammatory cytokines IL-1β and TNF-α in THP-1-derived macrophages. After 4 and 24 hours incubation, LPS-treated THP-1-derived macrophages showed elevated IL-8 (929 ± 124 and 2,328 ± 297 pg/ml, respectively), CCL4 (1,564 ± 3 pg/ml and 2,865 ± 72 pg/ml, respectively), IL-1β (83 ± 5 and 127 ± 8 pg/ml, respectively) and TNF-α, which was undetectable at 4 hours but reached 274 ± 21 pg/ml after 24 hours of incubation (Fig 4b).

Together, these data demonstrate that Csep1^P^ selectively induces chemokine production without triggering a canonical proinflammatory cytokine response, supporting the designation of a chemokine-dominant macrophage activation state.

### Csep1^P^ priming enhances macrophages proinflammatory responses to gut commensal bacterium *Escherichia coli*

**Fig 5:**
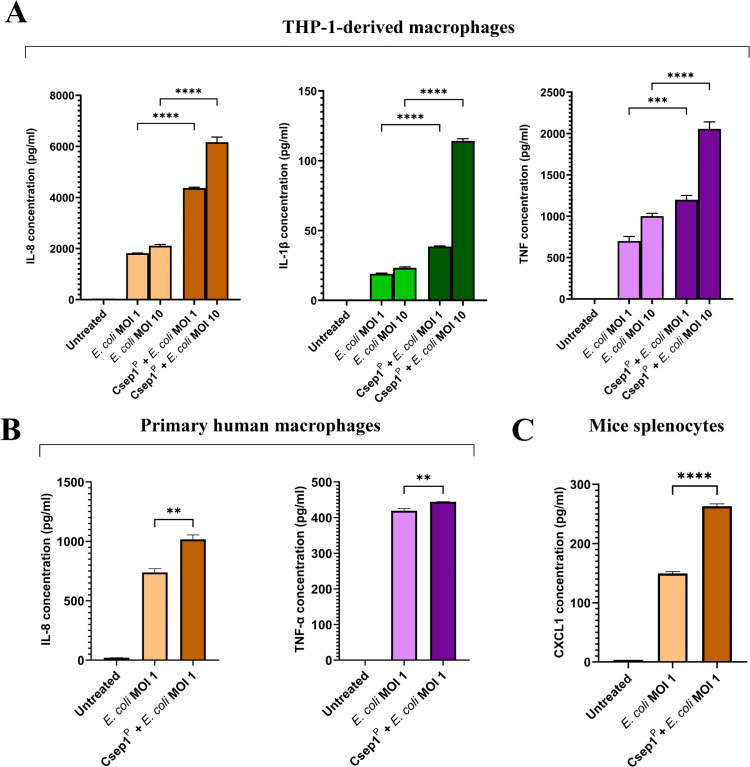
Effects of Csep1^P^ priming on production of chemokines and proinflammatory cytokines by macrophages in response to *E. coli* K12. The levels of chemokine IL-8 and proinflammatory cytokines IL-1β and TNF-α were measured using ELISA. **(a)** In THP-1-derived macrophages (10⁶ cells/ml), Csep1^P^ priming for 4 hours significantly increased IL-8, IL-1β, and TNF-α production following stimulation with *E. coli* K12 at both MOI 1 and MOI 10 for 2 hours, compared to non-primed macrophages. **(b)** In human primary macrophages (3.3 × 10⁵ cells/ml), Csep1^P^ priming for 4 hours significantly increased IL-8 and TNF-α production following stimulation using *E. coli* K12 at MOI 1 for 2 hours. IL-1β was below the limit of detection. Statistical analysis was performed using one-way analysis of variance (ANOVA) with Tukey’s post hoc test. Bars represent the mean of triplicate experiments ± SEM. **P < 0.01, ***P < 0.001, ****P < 0.0001. MOI: multiplicity of infection.

To assess the functional consequences of Csep1^P^-mediated M1-chem state activation, we evaluated the responses of both THP-1-derived and primary human macrophages to gut bacterium *E. coli* strain K12 by measuring IL-8, IL-1β, and TNF-α production. Our data showed that Csep1^P^-primed macrophages mounted a stronger proinflammatory response to *E. coli* (Fig 5).

Csep1^P^-primed THP-1-derived macrophages produced significantly higher levels of IL-8, IL-1β, and TNF-α upon stimulation with *E. coli* strain K12 for 2 hours at both multiplicities of infection (MOIs) of 1 and 10, compared to unprimed cells: IL-8: 4,369 ± 33.2 and 6,165 ± 197.8 pg/ml versus 1,818 ± 11.2 and 2,111 ± 53.1 pg/ml (*P* < 0.0001; Fig 5a); IL-1β: 38.6 ± 0.4 and 114.2 ± 1.7 pg/ml versus 19 ± 0.5 and 23.3 ± 0.6 pg/ml (*P* < 0.0001; Fig 5a); TNF-α: 1,199 ± 52.4 and 2,057 ± 84.7 pg/ml versus 700.7 ± 54.7 and 1,002 ± 33.2 pg/ml (*P* < 0.001 and *P* < 0.0001, respectively; Fig 5a).

Similar effects were observed in human primary macrophages. Csep1^P^-primed macrophages produced significantly more IL-8 and TNF-α in response to *E. coli* (MOI 1) than unprimed controls (1,017 ± 38.1 and 444.4 ± 0.3 pg/ml versus 739.4 ± 30.8 and 419.5 ± 5.7 pg/ml in controls; *P* < 0.01; Fig 5b). However, IL-1β was below the limit of detection in primary macrophage supernatants following *E. coli* stimulation for 2 hours, which is most likely due to the lower cell numbers used.

### Delta like canonical Notch ligand 4 (*DLL4)* silencing reduces CCL4 and TNF-α production in Csep1^P^-primed macrophages following *E. coli* challenge

**Fig 6:**
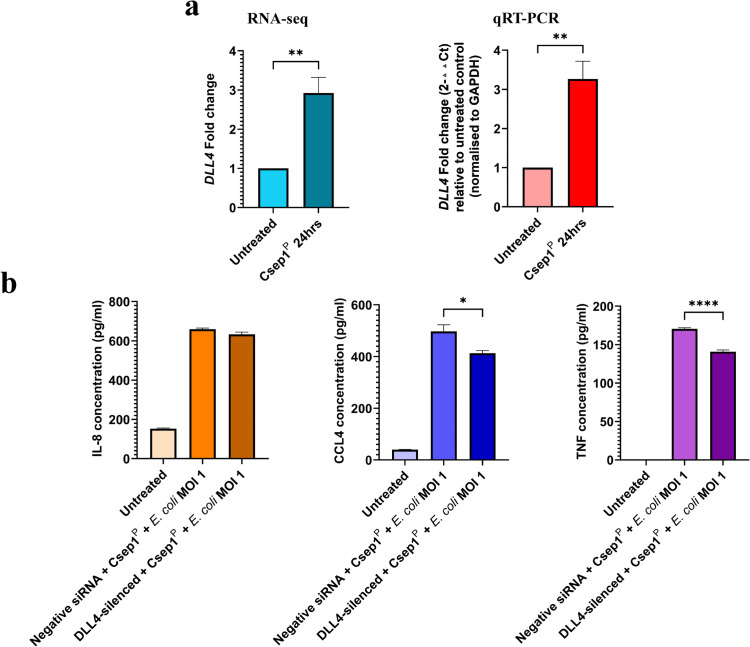
Upregulation of *DLL4* expression by Csep1^P^ in macrophages and its role in regulating macrophage response to *E. coli.* The change in *DLL4* gene expression in THP-1-derived macrophages induced by Csep1^P^ was measured using qRT-PCR. The levels of chemokines IL-8 and CCL4, and proinflammatory cytokines IL-1β and TNF-α in the supernatants of Csep1^P^-primed THP-1-derived macrophages with and without *DLL4* silencing were measured using ELISA. **(a)** Csep1^P^ induced significant upregulation of *DLL4* gene expression, demonstrated by both RNA-seq and qRT-PCR. Statistical significance was determined by two-tailed unpaired t-test. **(b)** Silencing *DLL4* gene expression significantly decreased the production of CCL4 and TNF-α in response to *E. coli* strain K12 following 2 hours incubation, but not IL-8. IL-1β was below the limit of detection. Statistical significance was determined by one-way analysis of variance (ANOVA) with Tukey’s post hoc test. Bars represent the mean of triplicate experiments ± SEM. *P < 0.05, **P < 0.01, ****P < 0.0001. MOI: multiplicity of infection.

Transcriptomic analysis using RNA-seq showed that Csep1^P^ significantly upregulated *DLL4* gene expression in THP-1-derived macrophages (Fig 6a and Table 1). We validated this by quantitative real-time PCR (qRT-PCR), which showed a 3.3 ± 0.5-fold increase in *DLL4* expression after 24 hours of incubation with Csep1^P^ (*P* < 0.01; Fig 6a).

To further determine the role of DLL4 in the enhanced response of Csep1^P^-primed macrophages to *E. coli* stimulation, we silenced *DLL4* in THP-1-derived macrophages using small interfering RNA (siRNA) and confirmed knockdown by qRT-PCR (S7 Fig). Macrophages were primed with Csep1^P^ for 4 hours, followed by a 2-hour stimulation with *E. coli* K12 (MOI 1). *DLL4* silencing significantly reduced CCL4 and TNF-α production compared to negative controls (CCL4: 413 ± 17.3 pg/ml versus 497.3 ± 44 pg/ml; *P* < 0.01; TNF-α: 140.8 ± 2.3 pg/ml versus 170.5 ± 1.5 pg/ml; *P* < 0.0001; Fig 6b). In contrast, IL-8 production was not significantly affected (633.2 ± 19.6 pg/ml versus 659 ± 11.3 pg/ml; *P* > 0.05; Fig 6b), and IL-1β was below the limit of detection under these conditions.

## Discussion

In this study, we determined the structure of the *C. concisus* Csep1^P^ protein and investigated its immunomodulatory role.

Structural analysis revealed that Csep1^P^ adopts a novel α-helical fold incorporating an α/α-solenoid moiety (Fig 1). The solenoid region showed remote structural similarity to *H. pylori* HcpB and HcpC proteins, indicating shared evolutionary pressures favouring a conserved functional scaffold. Although the extra-solenoidal domain of Csep1^P^ is unique to this protein, the conservation of the solenoid fold across distantly related bacterial genera suggests this domain is functionally important for host-pathogen interactions [31]. While the precise biological functions of most solenoid Hcp proteins remain poorly understood, the evidence of positive selection targeting surface-exposed residues in Hcp proteins suggests they play active roles in modulating bacterial-host interactions. A previous study reported that *H. pyl*ori HcpA, but not HcpC, promoted THP-1 monocyte-to-macrophage differentiation [29]. The precedent of HcpA’s role in immune modulation prompted us to investigate whether Csep1^P^ similarly functions in host immune manipulation, albeit potentially through distinct mechanisms. Under the experimental conditions used, we did not detect evidence of differentiation based on transcriptional signatures or cell morphology (S2 Table and S3 Fig).

However, our investigation on the effects of Csep1^P^ on macrophages revealed interesting results. Csep1^P^ induced a novel activation state in THP-1-derived macrophages. The chemokine and cytokine gene expression profile of this state was distinct from that of classical M1 macrophages induced by LPS plus IFN-γ (Fig 3 and Tables 1 and 2) [30]. We designate this distinct Csep1^P^-induced state as M1-chem, reflecting its predominant upregulation of chemokine genes.

We further validated the Csep1^P^-induced M1-chem state identified by transcriptomic analysis at the protein level by measuring representative chemokines and cytokines associated with M1-macrophage in both THP-1-derived and primary human macrophages. Csep1^P^ stimulation led to increased secretion of chemokines IL-8 and CCL4, but not proinflammatory cytokines IL-1β and TNF-α (Fig 4). This protein expression profile was consistent with the transcriptomic data, except for IL-1β. Although Csep1^P^ upregulated *IL1B* expression following 24 hours incubation, it did not increase IL-1β secretion (Figs 3 and 4a and Table 1). We speculate that Csep1^P^ alone does not provide the necessary signals required for inflammasome activation to cleave pro-IL-1β into its mature IL-1β form for detectable secretion [32]. In contrast, LPS stimulation induced robust secretion of IL-8, CCL4, IL-1β, and TNF-α (Fig 4b). Since chemokines such as IL-8 and CCL4 are potent immune cell recruiters [33], these findings suggest that Csep1^P^ may promote immune cell recruitment prior to overt inflammation, thereby reshaping the immune landscape.

Macrophage polarisation is increasingly recognised as a continuum rather than a M1 and M2 classification, with multiple intermediate or context-dependent activation states described [3]. In this context, the Csep1^P^-induced M1-chem state represents a non-canonical, chemokine-dominant macrophage activation state that is distinct from classical M1 polarisation. The selective induction of chemokines in the absence of strong proinflammatory cytokine secretion suggests that Csep1^P^ does not directly trigger full inflammatory activation but instead establishes a primed macrophage state poised for enhanced responses to secondary microbial stimulation, while also promoting immune cell recruitment. Indeed, in our experiments, although Csep1^P^ alone did not directly induce proinflammatory cytokine secretion, it altered macrophage responsiveness to commensal *E. coli*. Both THP-1-derived and human primary macrophages primed with Csep1^P^ exhibited significantly increased secretion of IL-8 and TNF-α upon exposure to *E. coli* strain K12 (Fig 5), supporting the role of Csep1^P^ in promoting exaggerated proinflammatory response to other gut bacteria.

Crohn’s disease predominantly affects the terminal ileum [34]. Despite the introduction of numerous anti-inflammatory therapies, relapse rates remain high, possibly because current treatments do not address microbial triggers. Each day, approximately 1 – 1.5 litres of saliva pass from the oral cavity to the intestines, providing a route for the repeated translocation of potential Crohn’s disease triggers, such as *C. concisus*, to the gastrointestinal tract. Virulent *C. concisus* strains can invade intestinal epithelial cells, induce epithelial cell death and produce proinflammatory secondary metabolites [35]. The pathogenesis of IBD is increasingly recognised to result from inappropriate immune responses to commensal gut bacteria, however the exact triggers remain unclear [36]. Our finding that Csep1^P^ activates macrophage to a M1-chem state and enhances macrophage proinflammatory responses to *E. coli* strain K12 suggests that virulent *C. concisus* strains may act as sensitisers by secreting immunomodulatory molecules such as Csep1^P^. This, in turn, could reshape the landscape of the mucosal immune system and reprogram macrophages to mount heightened proinflammatory responses to gut bacteria, thereby triggering the initiation or relapse of gut inflammation in Crohn’s disease.

A previous study by Nielsen *et al.* did not find an association between intestinal infection with *C. concisus* and an increased risk of subsequent development of IBD [18]. Our findings in this study raise the possibility that strain-specific factors, such as Csep1 expression, may be important, and that future studies assessing infection with Csep1-positive *C. concisus* strains are needed to clarify their potential role in Crohn’s disease.

The *DLL4* gene was the only signalling-related gene upregulated by Csep1^P^ (Fig 6a and Table 1). DLL4, a ligand of the Notch signalling pathway, has been shown to promote proinflammatory macrophage activation and to influence T-cell differentiation, particularly toward Th1 and Th17 lineages [37–39]. To assess its role in Csep1^P^-mediated macrophage responses, *DLL4* expression was silenced in THP-1-derived macrophages (S7 Fig). *DLL4* silencing significantly reduced secretion of CCL4 and TNF-α in Csep1^P^-primed macrophages following *E. coli* challenge (Fig 6b), indicating that DLL4-Notch signalling contributes to the enhanced macrophage inflammatory response induced by Csep1^P^. In contrast, IL-8 secretion was not affected by *DLL4* silencing (Fig 6b), likely reflecting the involvement of additional receptors and signalling pathways in *E. coli*-induced macrophage activation. Beyond its role in macrophages, DLL4 expressed by antigen-presenting cells has been reported to modulate T-cell responses through cell-cell Notch signalling interactions [40]. In the intestinal mucosa, such macrophage and T cell crosstalk may contribute to the amplification of Th1/Th17-type immune responses characteristic of Crohn’s disease [40]. Although direct effects on T cells were not examined in this study, the induction of DLL4 by Csep1^P^ suggests a potential mechanism by which *C. concisus*-derived molecules may influence both innate and adaptive immune responses through macrophage-mediated signalling.

In conclusion, our study demonstrates that the structurally unique Csep1^P^ induces a chemokine-dominant inflammatory state in macrophages and reprograms macrophage to enhance proinflammatory response to *E. coli*. These findings provide a potential mechanistic rationale for the role of virulent *C. concisus* strains in Crohn’s disease pathogenesis and identify Csep1^P^ as a potential therapeutic target.

## Materials and methods

### Determination of Csep1^P^ protein structure

#### Protein crystallisation and data collection.

Native crystals of recombinant Csep1^P^ (lacking the N-terminal signal peptide) were grown using the hanging-drop method as previously described [20]. The crystals belonged to space group *P*6_4_, with unit cell dimensions a = b = 85.8 Å, c = 55.2 Å, and contained one molecule per asymmetric unit. The platinum derivative was obtained by soaking crystals overnight in 1 mM potassium hexabromoplatinate. To perform data collection at cryogenic temperatures, crystals were loop-mounted in a cryoprotecting solution containing 36% w/v PEG 4000, 100 mM ammonium acetate, 80 mM sodium acetate trihydrate (pH 4.6), and 10% (v/v) glycerol, then flash-cooled by plunging into liquid nitrogen. Native X-ray diffraction data and SAD data for the platinum derivative (λ = 1.071 Å for both) were collected to 1.4 Å resolution on the MX2 beamline at the Australian Synchrotron. All data were processed and scaled using *XDS* [41] and *AIMLESS* [42] from the Collaborative Computational Project, Number 4 (*CCP4*) suite [43]. Data collection and processing statistics are summarised in S3 Table.

#### Structure determination.

Autosol [44] from the PHENIX software suite [45] was used to locate the platinum sites and build an initial partial model, comprising 173 residues (R = 0.239, R_free_ = 0.241). The model was then manually completed using Coot [46] and refined to R = 0.125, R_free_ = 0.156 using REFMAC [47], with waters placed by ARP/WARP [48]. This model was used for phasing the data for the native crystal by molecular replacement. The final model of Csep1^P^ (refined to R = 0.126, R_free_ = 0.167), contains all the expected amino acid residues (22–222) and 225 water molecules. Refinement statistics and stereochemistry are shown in S4 Table. All the non-glycine residues lie within permitted regions of the Ramachandran plot, with 99% in the most favoured regions. Representations of crystal structures were prepared using PYMOL (The PyMOL Molecular Graphics System, Version 2.0 Schrödinger, LLC) and UCSF Chimera [27].

### Csep1^P^ protein production and experimental optimisation

The expression vector for recombinant Csep1^P^ protein was based on the *csep1* gene sequence from the pICON plasmid of *C. concisus* strain P2CDO4 [19], minus the N-terminal signal peptide. Protein expression and purification were performed by GenScript (NJ, USA; S8 Fig). Protein identity was confirmed by mass spectrometry at the Mark Wainwright Analytical Centre, University of New South Wales, and no LPS was detected in the sample. Prior to incubation with macrophages, Csep1^P^ was sterilised by filtering through a 0.22 µm filter (Merck Millipore). Preliminary experiments were conducted to determine the ability of Csep1^P^ to induce responses in macrophages, and to optimise protein concentration and incubation times. Based on the results, 10 μg/ml of Csep1^P^ and 24-hour incubation were used for RNA-seq, while 10 μg/ml of Csep1^P^ and 4-hour incubation were used for priming experiments (S5 Table).

### Cells used in this study

The human monocytic leukaemia cell line THP-1 (ATCC No. TIB-202) was differentiated into macrophages using 10 nM phorbol 12-myristate 13-acetate (PMA; Sigma-Aldrich, NSW, AU) for 2 days as previously described [49]. Both undifferentiated and differentiated THP-1 cells were used. Primary macrophages were prepared from PBMCs obtained from a healthy individual and differentiated using 50 ng/ml recombinant human macrophage colony-stimulating factor (M-CSF; Sigma-Aldrich) for 7 days [50]. All cell types were cultured in RPMI 1640 medium (Thermo Fisher Scientific, CA, USA) supplemented with 10% foetal bovine serum (FBS; Cytiva, MA, USA), 100 U/ml penicillin, and 100 µg/ml streptomycin (Thermo Fisher Scientific), which we refer to as complete RPMI medium. Cells were maintained at 37°C in a humidified incubator with 5% CO_2_.

Macrophage differentiation was confirmed by quantifying expression of colony-stimulating factor 1 receptor (*CSF1R*) using qRT-PCR (Forward primer: GCTGCCTTACAACGAGAAGTGG; Reverse primer: CATCCTCCTTGCCCAGACCAAA; OriGene; SKU: HP208389). On top of gene expression change, the cell morphology of macrophages was also confirmed using light microscopy and detection of CD11b and CD68 expression by fluorescence microscopy [51].

### Examination of the effects of Csep1^P^ on THP-1 monocyte differentiation by fluorescence staining

THP-1 monocytes were seeded on coverslips in a 12-well plate at a concentration of 5 × 10^5^ cells/ml in complete RPMI medium with Csep1^P^ (10 μg/ml), with fresh Csep1^P^ added every 24 hours for 3 days. THP-1 monocytes incubated with 10 nM PMA were used as a positive control. THP-1 monocytes without treatment were used as an untreated control. The THP-1 cells were fixed with 3.6% paraformaldehyde for 15 minutes, permeabilised with 0.1% triton for 10 minutes, and blocked with 1% bovine serum albumin (BSA) for 1 hour. The filamentous actin (F-actin) and nuclei were then stained with Alexa Fluor 488 phalloidin (Cell Signaling Technology, MA, USA; Cat. no. 8878S) and Hoechst 33342 (Invitrogen, CA, USA), respectively. The cells were mounted onto glass slides and examined using a fluorescent microscope (Olympus BX61; Olympus, Tokyo, Japan) with FITC (Excitation wavelength: 480 nm; Emission wavelength: 520 nm) and DAPI (Excitation wavelength: 365 nm; Emission wavelength: 430 nm) filters to visualise the F-actin and nucleus, respectively.

### RNA-seq and transcriptomic analysis

Global gene expression in response to Csep1^P^ protein in naïve M0 macrophages was examined using RNA-seq. THP-1 monocytes and THP-1-derived macrophages were treated with Csep1^P^ (10 μg/ml) in RPMI medium without FBS and antibiotics for 24 hours. Untreated THP-1-derived macrophages serve as the negative control. Following incubation, total RNA was extracted from THP-1-derived macrophages using the ISOLATE II RNA Mini Kit (Bioline, NSW, Australia; Cat. no. BIO-52072) and submitted to the Australian Genome Research Facility Ltd for RNA-seq. Experiments were conducted in triplicate.

RNA-seq data were analysed as previously described [52]. Briefly, the quality of raw sequencing reads was assessed using FastQC (version 0.11.8) (http://www.bioinformatics.babraham.ac.uk/projects/fastqc/). Adapter sequences and low-quality bases were trimmed using Trimmomatic (version 0.38), with a minimum Phred score of 3 for both leading and trailing bases, and a sliding window of 4:15. Reads shorter than 30 bp following trimming were discarded [53]. Trimmed reads were aligned to the human reference genome GRCh38.p14 (GenBank ID: GCA_000001405.29) using HISAT2 (version 2.1.0) [54]. The resulting SAM files were converted to BAM format using SAMtools (version 1.11) [55], and gene-level quantification was performed using featureCounts from the Subread package (version 2.0.1) [56]. Differentially expressed genes (DEGs) between Csep1^P^-primed and untreated control samples were identified using the DESeq2 package (version 1.36.0) with default normalisation. Genes were considered significantly differentially expressed if they met the thresholds of adjusted *P*-value < 0.05 and log_2_ fold change ≤ -1 or ≥ 1 [57]. The list of DEGs was submitted to the Enrichr database for GO enrichment analysis to identify associated biological processes [58–60].

### Comparison of gene expression of chemokines and proinflammatory cytokines induced by Csep1^P^ and LPS

The chemokine and proinflammatory cytokine genes upregulated by Csep1^P^ in this study were compared with previously reported M1-macrophage associated cytokines induced by LPS plus IFN-γ [30]. Genes that were significantly differentially expressed, with the thresholds of adjusted *P*-value < 0.05 and log_2_ fold change ≤ -1 or ≥ 1, were included for comparison.

### Measurement of secreted cytokines by macrophages in response to Csep1^P^

To validate the expression of upregulated chemokines and proinflammatory cytokines induced by Csep1^P^ in the transcriptomic analysis, we measured the protein levels of two representative chemokines including IL-8 and CCL4, and two representative proinflammatory cytokines including IL-1β and TNF-α. The selection of these cytokines was based on their association with M1 macrophage and IBD [61–63].

THP-1-derived macrophages were seeded in 6-well plates at a density of 10^6^ cells/ml and incubated with Csep1^P^ (10 μg/ml) in RPMI medium without FBS and antibiotics for 4 hours and 24 hours; untreated cells served as a negative control. Supernatants were collected and chemokine and cytokine levels were measured using commercially available ELISA kits (Invitrogen; Cat. no. IL-8: CHC1301, CCL4: 88–7034, IL-1β: CHC1213, TNF-α: CHC1753). Human primary macrophages (3.3x10^5^ cells/ml) were also used to validate the 24-hour Csep1^P^ incubation results. All measurements were conducted in triplicate. LPS (from *E. coli* O55:B5; 100 ng/ml; Sigma-Aldrich; Cat. no. L6529) incubated with THP-1-derived macrophages for 4 hours and 24 hours was used as a positive control for measurement of IL-8, CCL4, IL-1β, and TNF-α [64].

### Investigating the impact of Csep1^P^ on macrophage response to commensal gut bacteria

To investigate the potential role of Csep1^P^ protein in modulating macrophage function, we compared the production levels of IL-8, IL-1β, and TNF-α in THP-1-derived macrophages following exposure to microbial stimulation with the commensal *E. coli* strain K12, with and without Csep1^P^ priming. Briefly, THP-1-derived macrophages (10^6^ cells/ml) were incubated in RPMI medium with or without Csep1^P^ (10 μg/ml). After 4 hours, cells were washed with Dulbecco’s phosphate-buffered saline (DPBS), resuspended in RPMI medium without FBS and antibiotics, and stimulated with heat-killed *E. coli* K12 at MOI 1 or 10. Primary macrophages (3.3x10^5^ cells/ml) were also used to validate the MOI 1 results. After a 2-hour incubation, supernatants were collected, and IL-8, IL-1β, and TNF-α levels were measured using ELISA kits. All measurements were conducted in triplicate.

### Investigation of the potential involvement of DLL4 in Csep1^P^ upregulated macrophage response to *E. coli*

Our transcriptomic analysis identified upregulation of the *DLL4* gene, we confirmed it using qRT-PCR (Forward primer: AACTACTGCACCCACCACT, Reverse primer: GCCATCCTCCTGGTCCTTACA) [65]. We then examined whether silencing the *DLL4* gene expression impacts the Csep1^P^-upregulated macrophage response to *E. coli.* The *DLL4* gene encodes a ligand for Notch receptors [37].

For silencing *DLL4* gene expression, THP-1-derived macrophages were seeded in 6-well plates at a density of 10^6^ cells/ml and transfected with 40 nM of two siRNA sets (SASI_Hs01_00174509 and SASI_Hs02_00352665; Sigma-Aldrich) for 16 hours. Transfection was carried out using Lipofectamine RNAiMAX reagent (Thermo Fisher Scientific) diluted in Opti-MEM Reduced Serum Medium (Thermo Fisher Scientific) [66]. MISSION GAPDH SiRNA (SASI_Hs01_00140986; Sigma-Aldrich) and MISSON Universal Negative Control #1 (Sigma-Aldrich, Cat. No. SIC001) were used as positive and negative controls, respectively. The efficiency of *DLL4* gene silencing was validated using qRT-PCR [67].

*DLL4*-silenced cells were washed with DPBS, resuspended in RPMI medium without FBS and antibiotics, and incubated with Csep1^P^ (10 μg/ml) for 4 hours. Cells were then washed again with DPBS and incubated with heat-killed *E. coli* K12 at MOI 1 in RPMI medium without FBS and antibiotics for 2 hours. Supernatants were collected and levels of IL-8, IL-1β, CCL4, and TNF-α were measured using ELISA kits. All measurements were conducted in triplicate.

### Statistical analysis

All measurements for RNA-seq and ELISAs were conducted in triplicate. For comparisons involving multiple treatment groups and the control group, statistical significance was assessed using one-way analysis of variance (ANOVA) followed by Tukey’s post hoc test. For comparisons between a single treatment group and the control, a two-tailed unpaired t-test was used. All statistical analyses were performed using GraphPad Prism (v9.3.1). *P* < 0.05 was considered statistically significant. All data were expressed as mean of triplicate experiments ± standard error of the mean (SEM), unless otherwise stated.

## Supporting information

S1 Fig Structure superpositions of *C. concisus* Csep1^P^ protein with *H. pylori* HcpB and HcpC. The superpositions of the solenoid moiety (red) of the structure of *C. concisus* Csep1^P^ with the crystal structures of *H. pylori* HcpB (PCD ID 1klx) and HcpC (PDB ID 1ouv), highlighting the structural conservation of the internal disulfide bonds (shown as spheres) stabilising the α-α-hairpins in these proteins.
(TIF)

S2 Fig Conserved residue clusters on the surface of the solenoid moiety of Csep1^P^. Stereo diagram showing the C-terminus lodged within the concave groove of the solenoid moiety. The side chains of highly variable residues at positions 203 and 209 (cyan) are accommodated within highly conserved (magenta) pockets on the concave surface of the solenoid.
(TIF)

S3 Fig Fluorescence microscope images and Gene Ontology (GO) analysis of THP-1 monocytes incubated with Csep1^P^ or PMA for 72 hours. **(a)** THP-1 monocytes incubated with media only (untreated), Csep1^P^, or PMA were observed after 72 hours. Cell nucleus and F-actin were stained with Hoechst 33342 and Alexa Fluor 488 phalloidin and visualised using DAPI and FITC filters, respectively. Incubation with Csep1^P^ resulted in slight increase in cell size and F-actin aggregation when compared with the untreated cells. PMA-treated THP-1 monocytes were used as a positive control for macrophage-like differentiation. Both Csep1^P^-treated and untreated THP-1 monocytes showed adherence to the cover slip. Scale bars for 20× and 100 × magnifications represent 50 μm and 10 μm, respectively. **(b)** Enriched GO terms of the 15 significantly upregulated genes, sorted according to *P-*value. The top three enriched terms were “regulation of defence response”, “natural killer cell degranulation”, and “neutrophil degranulation”. **(c)** Enriched GO terms of the 32 significantly downregulated genes, sorted according to *P-*value. The top three enriched terms were “myeloid cell activation involved in immune responses”, “protein K48-linked deubiquitination”, and “protein K63-linked deubiquitination”.
(TIF)

S4 Fig Principal component analysis (PCA) of RNA-seq data. A total of 6 samples were included in the analysis, with red dots representing Csep1^P^-treated and blue dots representing untreated control THP-1-derived macrophages. The plot shows clear separation between control and treatment groups, indicating significant changes in global gene expression in THP-1-derived macrophages after 24-hr incubation with Csep1^P^. Gene counts were log_2_-transformed and normalised prior to PCA. The PCA plot was generated using the ggplot2 package.
(TIF)

S5 Fig Heatmap of differentially expressed genes in Csep1^P^-treated THP-1-derived macrophages. The heatmap shows 101 genes that were differentially expressed (*P* < 0.05; log_2_ fold change ≤ -1 or ≥ 1). The gene read counts of the experimental triplicates obtained using featureCounts from the Subread package (version 2.0.1) were log2-normalised and expressed in a colour scheme. Heatmap was generated using the pheatmap package.
(TIF)

S6 Fig Confirmation of primary macrophage differentiation using microscopy and qRT-PCR. **(a)** Light microscopy image (40×) showing that primary macrophages differentiated from peripheral blood mononuclear cells (PBMCs) using macrophage colony-stimulating factor (M-CSF), display a characteristic elongated phenotype. **(b)** M-CSF treatment significantly upregulated *CSF1R* gene expression in primary macrophages (1.6 ± 0.04-fold change, *** = *P* < 0.001). Fold change was calculated using the comparative threshold cycle CT (2-ΔΔCT) method, with target gene expression normalised to the housekeeping gene GAPDH and calculated relative to the untreated control. Statistical significance was assessed using two-tailed unpaired t-test. Bars represent the mean of triplicate experiments ± SEM. *** = *P* < 0.001. **(c)** Immunofluorescence staining of cell nuclei (Hoechst 33342, DAPI filter), CD11b (anti-CD11b antibody, FITC filter), and CD68 (anti-CD68 antibody, CY5 filter), visualised at 100 × magnification. Treatment of macrophages with M-CSF resulted in increased fluorescence intensity for both CD11b and CD68 compared to untreated cells. Scale bars represent 10 μm.
(TIF)

S7 Fig Confirmation of *DLL4* gene silencing in THP-1-derived macrophages with and without Csep1^P^ and *E. coli* stimulation. *DLL4* gene expression in *DLL4*-silenced THP-1-derived macrophages was measured by qRT-PCR to validate the gene silencing efficacy of the transfected siRNA. **(a)** *DLL4* silencing was confirmed by a significant reduction in *DLL4* gene expression, showing a 0.37 ± 0.03-fold change (*P* < 0.0001). **(b)** *DLL4* silencing significantly reduced *DLL4* expression in Csep1^P^-primed THP-1-derived macrophages incubated with *E. coli*, showing a 62.6 ± 5.5-fold and 34.1 ± 4.7-fold change for the siRNA negative control and *DLL4*-silenced cells, respectively (*P* < 0.01). *DLL4* gene fold change is shown relative to the untreated control and normalised to the housekeeping gene GAPDH. Statistical significance was assessed by two-tailed unpaired t-test for (a) and by one-way analysis of variance (ANOVA) with Tukey’s post-hoc test for (b). Bars represent the mean of triplicate experiments ± SEM. ** = *P* < 0.01, **** = *P* < 0.0001. MOI = multiplicity of infection.
(TIF)

S8 Fig Coomassie Blue-stained SDS-PAGE gel of recombinant *C. concisus* Csep1^P^ used for transcriptomic and cytokine production analyses in macrophages. The N-terminally His6-tagged protein was expressed in *E. coli* BL21 Star (DE3) and purified by GenScript. Its identity was confirmed by mass spectrometry. LPS was not detectable in the purified protein using the Pierce Chromogenic Endotoxin Quant Kit. BSA: bovine serum albumin.
(TIF)

S1 Table Results of the similarity search of Csep1^P^ against the protein structures deposited in the PDB. (XLSX)

S2 Table Differentially expressed genes in THP-1 monocytes after incubation with Csep1^P^ protein from transcriptomic analysis. (XLSX)

S3 Table X-ray data collection and processing statistics. (XLSX)

S4 Table Refinement statistics. (XLSX)

S5 Table Optimisation of Csep1^P^ dosage. (XLSX)
