# Supplementary figures and images for: Csep1P protein from Campylobacter concisus induces a chemokine-dominant inflammatory state in macrophages and enhances proinflammatory response to gut bacteria
Source: PLoS Pathog. 2026 Feb 13;22(2):e1013951. doi: 10.1371/journal.ppat.1013951 (PMC12904459; doi:10.1371/journal.ppat.1013951)

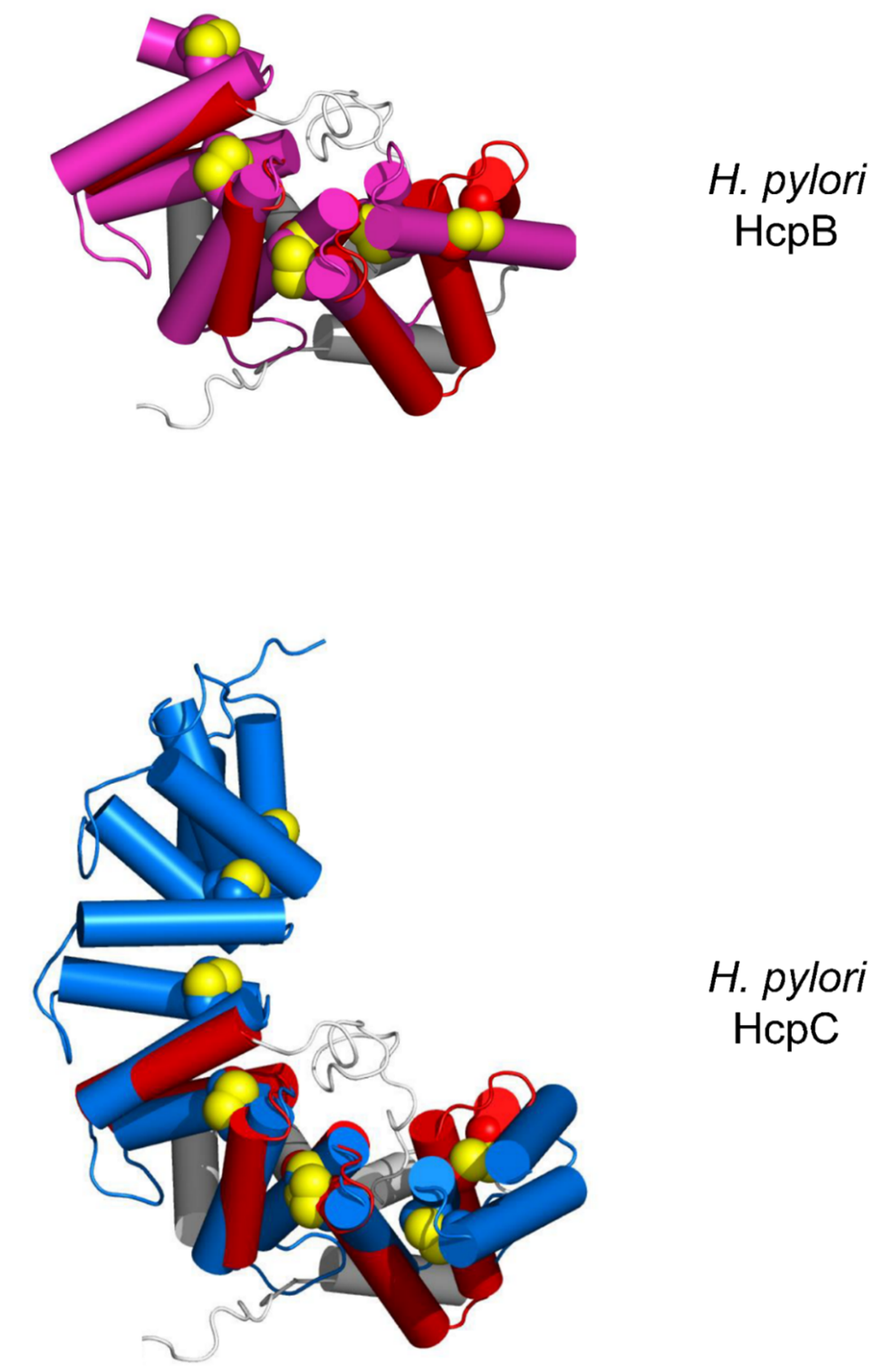

Supplement: S1 Fig — The superpositions of the solenoid moiety (red) of the structure of C. concisus Csep1P with the crystal structures of H. pylori HcpB (PCD ID 1klx) and HcpC (PDB ID 1ouv), highlighting the structural conservation of the internal disulfide bonds (shown as spheres) stabilising the α-α-hairpins in these proteins. (TIF) [file ppat.1013951.s001.tif]

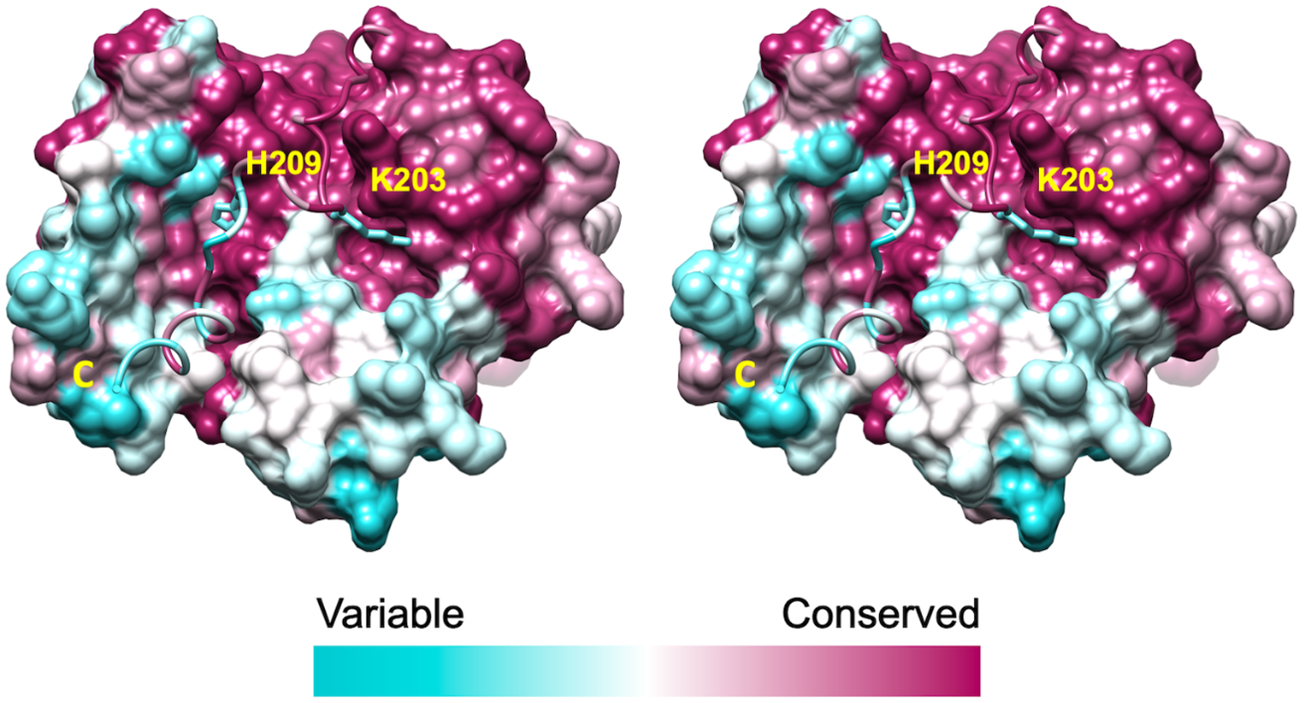

Supplement: S2 Fig — The side chains of highly variable residues at positions 203 and 209 (cyan) are accommodated within highly conserved (magenta) pockets on the concave surface of the solenoid. (TIF) [file ppat.1013951.s002.tif]

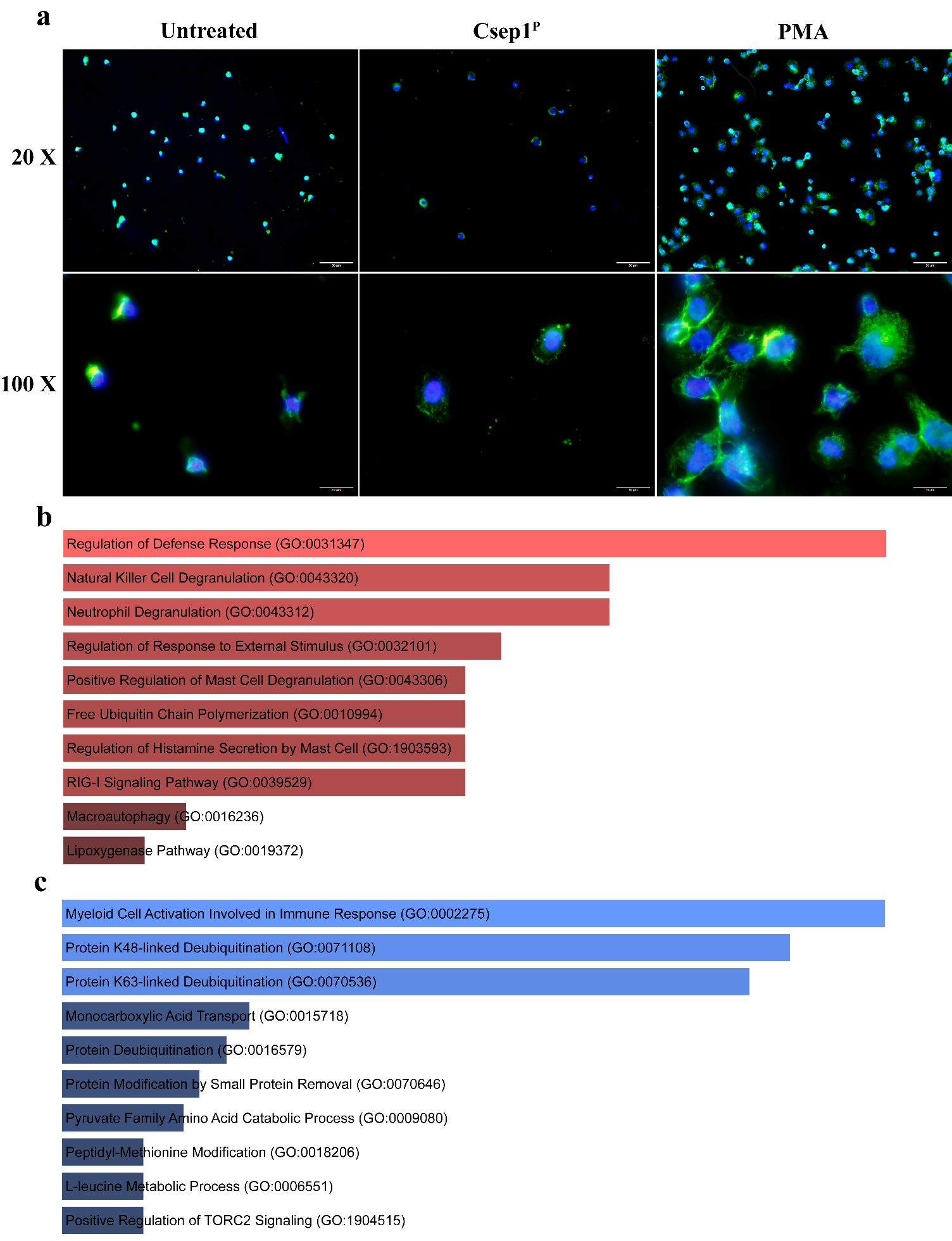

Supplement: S3 Fig — (a) THP-1 monocytes incubated with media only (untreated), Csep1P, or PMA were observed after 72 hours. Cell nucleus and F-actin were stained with Hoechst 33342 and Alexa Fluor 488 phalloidin and visualised using DAPI and FITC filters, respectively. Incubation with Csep1P resulted in slight increase in cell size and F-actin aggregation when compared with the untreated cells. PMA-treated THP-1 monocytes were used as a positive control for macrophage-like differentiation. Both Csep1P-treated and untreated THP-1 monocytes showed adherence to the cover slip. Scale bars for 20× and 100 × magnifications represent 50 μm and 10 μm, respectively. (b) Enriched GO terms of the 15 significantly upregulated genes, sorted according to P-value. The top three enriched terms were “regulation of defence response”, “natural killer cell degranulation”, and “neutrophil degranulation”. (c) Enriched GO terms of the 32 significantly downregulated genes, sorted according to P-value. The top three enriched terms were “myeloid cell activation involved in immune responses”, “protein K48-linked deubiquitination”, and “protein K63-linked deubiquitination”. (TIF) [file ppat.1013951.s003.tif]

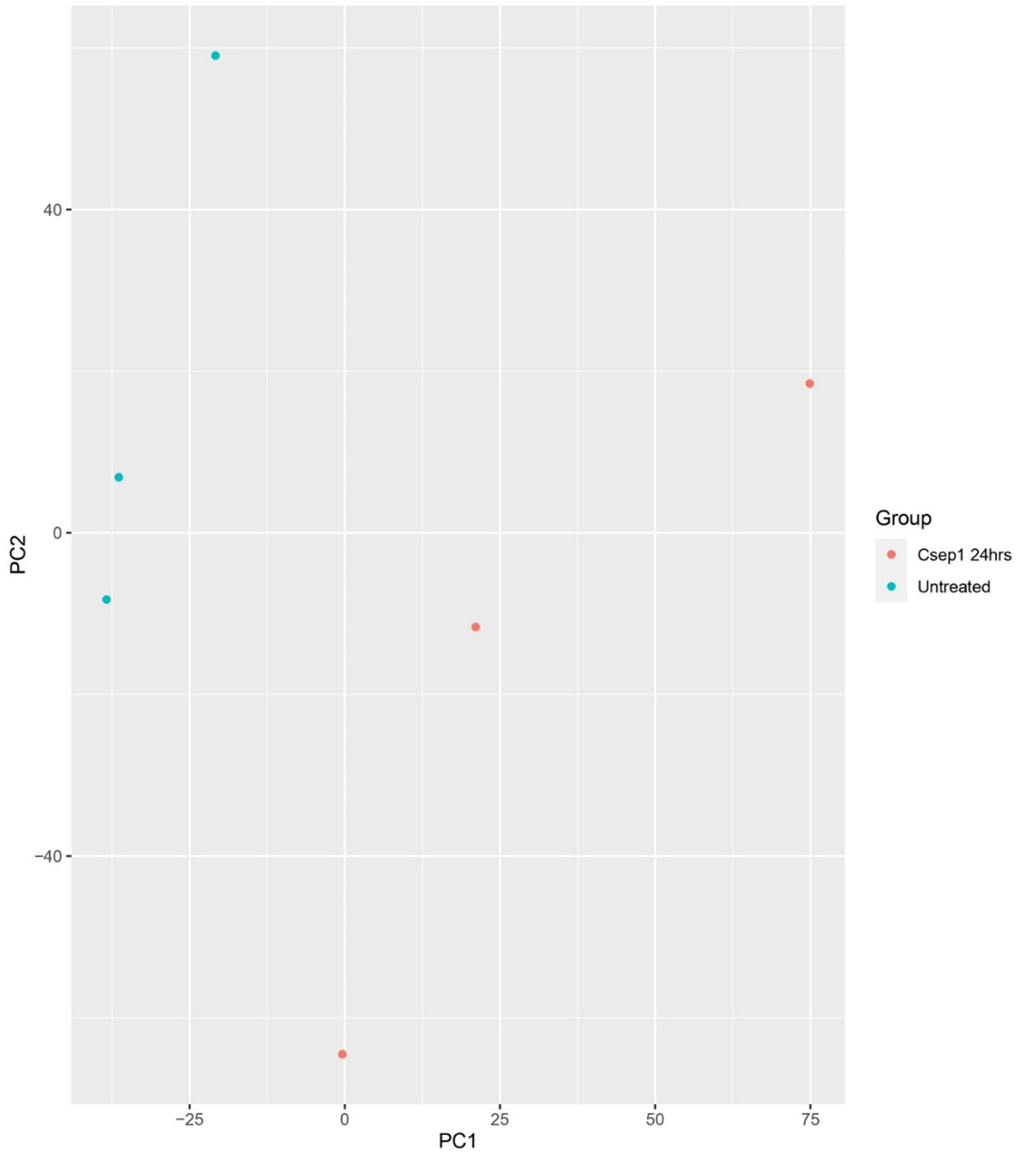

Supplement: S4 Fig — A total of 6 samples were included in the analysis, with red dots representing Csep1P-treated and blue dots representing untreated control THP-1-derived macrophages. The plot shows clear separation between control and treatment groups, indicating significant changes in global gene expression in THP-1-derived macrophages after 24-hr incubation with Csep1P. Gene counts were log2-transformed and normalised prior to PCA. The PCA plot was generated using the ggplot2 package. (TIF) [file ppat.1013951.s004.tif]

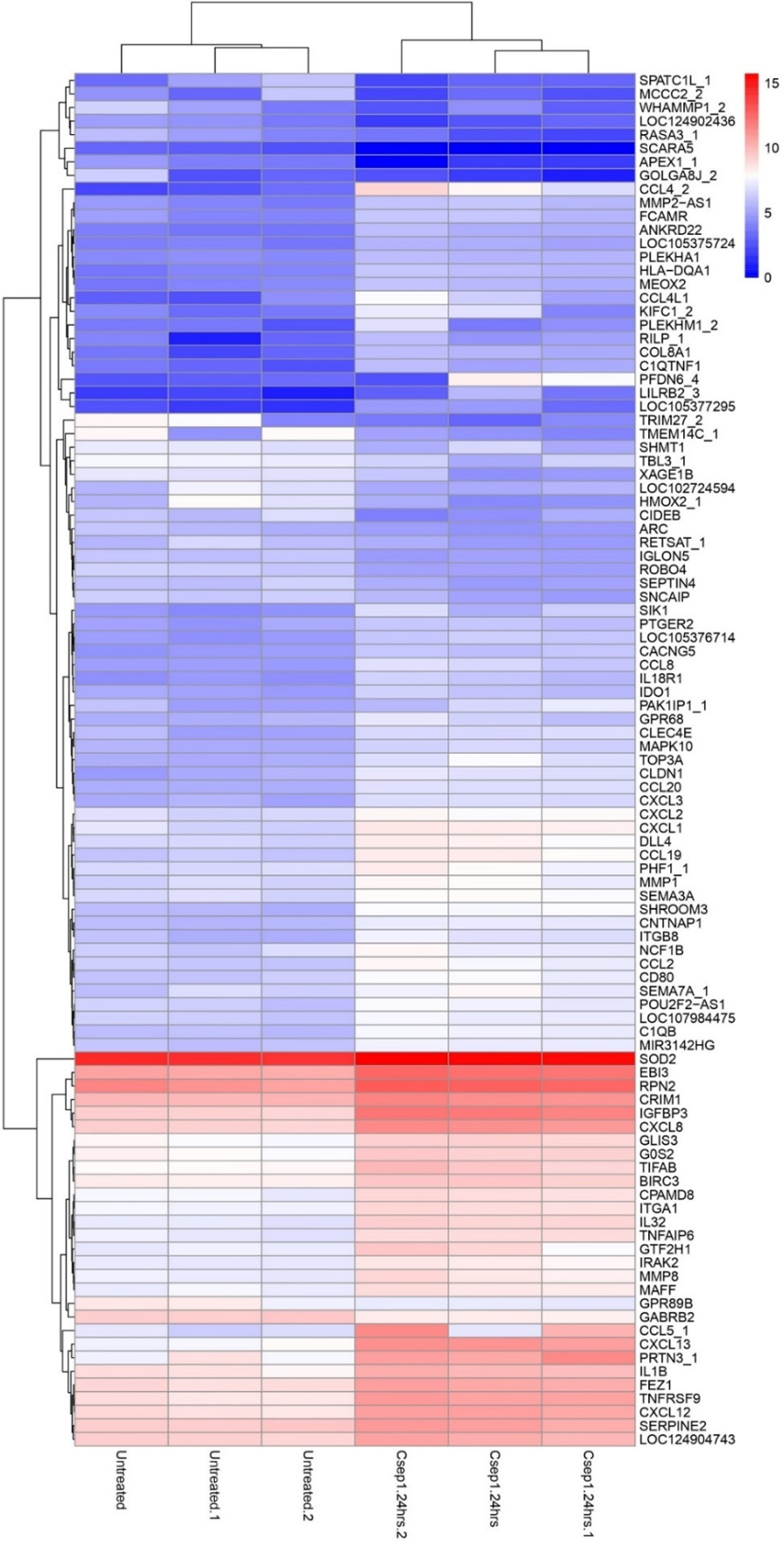

Supplement: S5 Fig — The heatmap shows 101 genes that were differentially expressed (P < 0.05; log2 fold change ≤ -1 or ≥ 1). The gene read counts of the experimental triplicates obtained using featureCounts from the Subread package (version 2.0.1) were log2-normalised and expressed in a colour scheme. Heatmap was generated using the pheatmap package. (TIF) [file ppat.1013951.s005.tif]

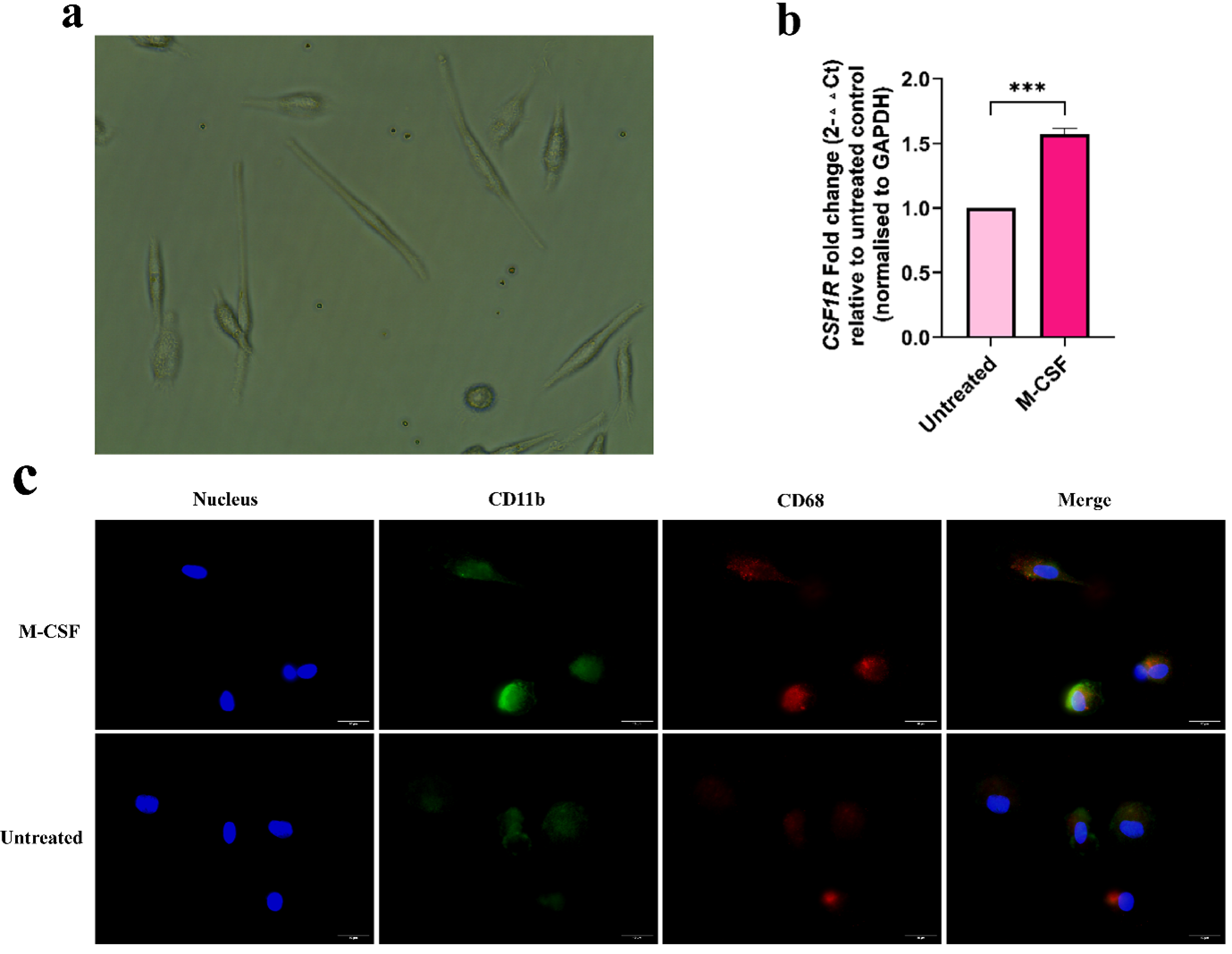

Supplement: S6 Fig — (a) Light microscopy image (40×) showing that primary macrophages differentiated from peripheral blood mononuclear cells (PBMCs) using macrophage colony-stimulating factor (M-CSF), display a characteristic elongated phenotype. (b) M-CSF treatment significantly upregulated CSF1R gene expression in primary macrophages (1.6 ± 0.04-fold change, *** = P < 0.001). Fold change was calculated using the comparative threshold cycle CT (2-ΔΔCT) method, with target gene expression normalised to the housekeeping gene GAPDH and calculated relative to the untreated control. Statistical significance was assessed using two-tailed unpaired t-test. Bars represent the mean of triplicate experiments ± SEM. *** = P < 0.001. (c) Immunofluorescence staining of cell nuclei (Hoechst 33342, DAPI filter), CD11b (anti-CD11b antibody, FITC filter), and CD68 (anti-CD68 antibody, CY5 filter), visualised at 100 × magnification. Treatment of macrophages with M-CSF resulted in increased fluorescence intensity for both CD11b and CD68 compared to untreated cells. Scale bars represent 10 μm. (TIF) [file ppat.1013951.s006.tif]

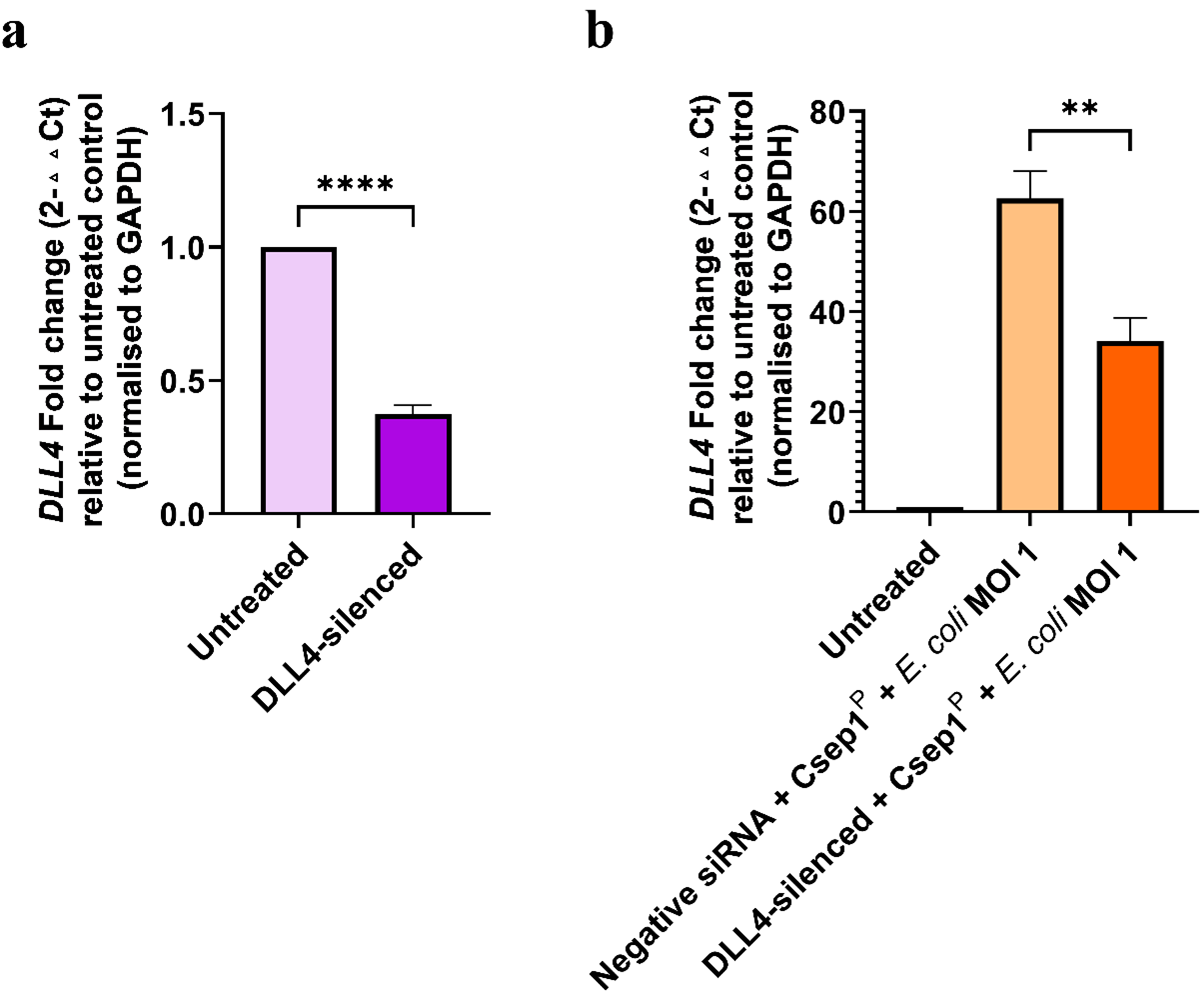

Supplement: S7 Fig — DLL4 gene expression in DLL4-silenced THP-1-derived macrophages was measured by qRT-PCR to validate the gene silencing efficacy of the transfected siRNA. (a) DLL4 silencing was confirmed by a significant reduction in DLL4 gene expression, showing a 0.37 ± 0.03-fold change (P < 0.0001). (b) DLL4 silencing significantly reduced DLL4 expression in Csep1P-primed THP-1-derived macrophages incubated with E. coli, showing a 62.6 ± 5.5-fold and 34.1 ± 4.7-fold change for the siRNA negative control and DLL4-silenced cells, respectively (P < 0.01). DLL4 gene fold change is shown relative to the untreated control and normalised to the housekeeping gene GAPDH. Statistical significance was assessed by two-tailed unpaired t-test for (a) and by one-way analysis of variance (ANOVA) with Tukey’s post-hoc test for (b). Bars represent the mean of triplicate experiments ± SEM. ** = P < 0.01, **** = P < 0.0001. MOI = multiplicity of infection. (TIF) [file ppat.1013951.s007.tif]

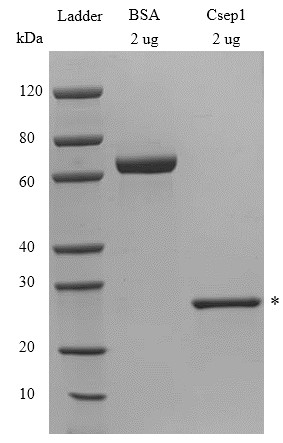

Supplement: S8 Fig — The N-terminally His6-tagged protein was expressed in E. coli BL21 Star (DE3) and purified by GenScript. Its identity was confirmed by mass spectrometry. LPS was not detectable in the purified protein using the Pierce Chromogenic Endotoxin Quant Kit. BSA: bovine serum albumin. (TIF) [file ppat.1013951.s008.tif]
